# Platelet-derived exosomal LINC00183 facilitate colorectal cancer malignant progression driven by histone lactylation through stabilizing ENO1

**DOI:** 10.1038/s41419-025-07914-4

**Published:** 2025-08-07

**Authors:** Guoqing Su, Jinghang Qian, Yi Wang, Yu Zhu, Yanyan Chen, Jingru Shen, Jiayuan Jiang, Yuepeng Cao, Nannan Wang, Xing Huang, Chengshuai Si, Xu Zhang, Peng Shao, Yongxia Ye, Yang Wang, Jun Bao, Liu Yang

**Affiliations:** 1 Department of Colorectal Surgery, The Affiliated Cancer Hospital of Nanjing Medical University & Jiangsu Cancer Hospital & Jiangsu Institute of Cancer Research, Nanjing, China; 2 Department of Medical Oncology, The Affiliated Cancer Hospital of Nanjing Medical University & Jiangsu Cancer Hospital & Jiangsu Institute of Cancer Research, Nanjing, China; 3 Gastrointestinal Surgery, Beijing Shijitan Hospital, Capital Medical University, Beijing, China; 4 Department of Pathology, The Affiliated Cancer Hospital of Nanjing Medical University & Jiangsu Cancer Hospital & Jiangsu Institute of Cancer Research, Nanjing, China

**Keywords:** Cancer metabolism, Post-translational modifications

## Abstract

Platelets play a critical role in tumor progression across various cancers. However, little is known about the molecular mechanisms and biological roles of exosomal long non-coding RNAs (lncRNAs) produced from platelets in colorectal cancer (CRC). Using RNA sequencing, we identified LINC00183 as the most upregulated lncRNA in platelet-derived exosomes (PLT-Exos) from CRC patients, compared to healthy individuals. Analysis of CRC tissue microarrays and TCGA-CRC patient data indicated that LINC00183 is often overexpressed in CRC, in association with advanced tumor stage and poor survival. Effective transfer of exosomal LINC00183 to human CRC cells was verified by FISH, western blotting, and RT-qPCR analyses. Co-incubation with PLT-Exos from CRC patients and overexpression of LINC00183 stimulated the proliferative and invasive capacities of CRC cells. RNA pull-down and RNA immunoprecipitation assays revealed that LINC00183 interacts with enolase 1 (ENO1). This interaction rescues ENO1 from ubiquitin-proteasome-mediated degradation by masking a critical K262 residue. Co-immunoprecipitation, western blotting, CUT&Tag, and gene knockdown and overexpression assays further revealed that the LINC00183-ENO1 interaction activates glycolysis in CRC cells, leading to lactate accumulation, H3K18 lactylation, and transcriptional upregulation of the oncogene GDF15. These results highlight LINC00183 as a possible therapeutic target for CRC.

## Introduction

One of the most prevalent cancers of the gastrointestinal tract is colorectal cancer (CRC) [1], standing as the third most prevalent cancer and a leading cause of cancer deaths around the world [2]. The 5-year survival rates for colorectal cancer (CRC) vary significantly between studies; the rates for stages I and II are 91% and 80%, respectively, while the rates for stages III and IV are 61.7% and 23.2% [3]. Approximately 42% of patients in stages II and III are alarmingly affected by local recurrence or distant metastasis [4]. Consequently, understanding the mechanisms driving CRC progression and discovering trustworthy biomarkers for early intervention is urgently needed.

There is strong evidence that tumor-associated platelets play a critical role in accelerating the development of cancer [5]. Platelets, acting as versatile agents in the process of tumorigenesis, constitute the largest and most extensive supply of tumor-promoting factors in the blood, emerging as significant biomarkers and diagnostic resources for multiple cancer types [6]. Platelet aggregation and degranulation, which lead to the release of pro-angiogenic factors from platelets, play a role in creating a pro-inflammatory and immune-suppressive tumor microenvironment, affecting tumor growth [7]. In various types of cancer, the interplay between platelet activation and coagulation is associated with disease progression, often leading to an unfavorable prognosis for patients [8].

Exosomes are tiny membrane-bound vesicles that cells release; they usually have a diameter of 30 to 150 nm and are essential in a variety of physiological and pathological circumstances. Exosomes primarily originate from multivesicular bodies and are released into the extracellular space following their fusion with the cell membrane [9]. Exosomes include proteins, lipids, nucleic acids, and other cargo components that allow them to control the biological activity of recipient cells after being secreted from host cells [10]. According to a growing amount of studies, exosome-mediated intercellular communication controls critical cellular functions including as immunological responses, antigen presentation, differentiation, and proliferation. It also affects the development, migration, and invasion of cancer cells [11].

Long non-coding RNAs, or lncRNAs, are RNA molecules that are longer than 200 nucleotides and do not encode proteins [12]. They are essential regulators of many biological processes, including transcriptional control and chromatin remodeling. LncRNAs interact with proteins, RNA, and DNA to affect biological processes; they frequently operate as molecular scaffolds or decoys [13]. It is well established that activated platelets release large quantities of exosomes [14]. Platelets are the primary source of exosomes in peripheral blood, accounting for approximately two-thirds of all circulating exosomes [15].

The potential contribution of platelet-derived exosomal lncRNAs to CRC progression remains unclear. To deal with this issue, we carried out RNA sequencing (RNA-seq) to analyze the differences in lncRNA expression in exosomes from platelet-rich plasma samples of CRC patients and healthy controls. We then concentrated on LINC00183, the most highly elevated lncRNA species in CRC samples, and described the processes behind its pro-tumorigenic effects on CRC using both in vitro and in vivo investigations. Our findings suggest that LINC00183 from platelet-derived exosomes may be a therapeutic target and prognostic marker in colorectal cancer.

## Materials and Methods

### Blood collection

Pathologically confirmed, newly diagnosed, and treatment-naive CRC patients were recruited at Jiangsu Cancer Hospital (Nanjing, China). Before the study began, written informed agreement was obtained from all participants, and the Ethics Committee of Nanjing Medical University (Nanjing, China) approved the study methodology. Blood samples were taken from patients and healthy volunteers at Jiangsu Cancer Hospital.

### Platelet preparation

Blood samples were processed within two hours of being collected, with 5 mL of whole blood drawn into ACD vacutainer tubes. By centrifuging at 400 × g for 10 minutes, platelet-rich plasma was acquired. To inhibit platelet activation, 100 ng of PGE2 (Cat. no. 5640, Sigma, USA) was introduced to the PRP. The platelets were then separated by centrifuging the PRP at 2000 g for 10 minutes. After collecting the supernatant for further exosome isolation, the platelet pellet was resuspended in 100 ng of PGE2 in Dulbecco’s phosphate-buffered saline (DPBS).

### Exosome purification

To separate exosomes released by platelets, the collected supernatant was centrifuged using an Optima SW 55 Ti rotor (Beckman Coulter, USA) at 26,500 *g* for 30 minutes at 4 °C. To pellet the exosomes, the supernatant was centrifuged for 70 minutes at 110,000 × *g*. The pellet underwent two more ultracentrifugation processes at 110,000 × g for 60 minutes each, with DPBS washes in between, after the supernatant was disposed of. The completed exosome pellet was stored at −80 °C for future use.

### Exosome characterization

The morphology of exosome particles was characterized using transmission electron microscopy (TEM; JEOL, Japan). Exosomal protein markers were assessed by western blotting. Using a ZetaView PMX 110 device (Particle Metrix, Germany) and OriginPro 8.5 software (OriginLab Co., USA), exosome yields were measured by nanoparticle tracking analysis (NTA).

### Exosome uptake assay

The PKH26 red fluorescent kit (Solarbio, USA) was used to label the exosomes. In short, the cleaned exosomes were gently combined with PKH26 after being resuspended in Dilution C reagent. To quench any unbound PKH26 dye, an equivalent volume of fetal bovine serum (FBS) was subsequently added. The PKH26-labeled exosomes were resuspended in culture media and utilized to treat cells following an ultracentrifuge (100,000 *g*, 2 hours, 4 °C). The uptake of exosomes in CRC cells was observed using a fluorescent microscope (Nikon, Japan).

### RNA fluorescence in situ hybridization (FISH)

GenePharma (Shanghai, China) generated the Cy3-labeled LINC00183 probe. As directed by the manufacturer, hybridization was carried out using a FISH detection kit (GenePharma, China). An epifluorescence microscope (Olympus, Tokyo, Japan) was used to take pictures after nuclei were stained with DAPI. Supplementary Table S1 contains detailed information about the probes used in this investigation.

### Immunofluorescence assay

The cells were fixed with 4% paraformaldehyde and then permeabilized with 0.1% Triton X-100. For blocking, a 2.5% BSA solution was employed. Following primary and secondary antibody incubation, pictures were taken with a Nikon epifluorescence microscope (Japan). DAPI was used to stain the nuclei.

### Whole-transcriptome sequencing of platelet-derived exosomes

An lncRNA microarray analysis of platelet-derived exosomes (PLT-Exos) was conducted at LC-BIO Co., Ltd (HangZhou, China). In summary, total exosomal RNA was extracted and purified from the platelets of four colorectal cancer patients and four age-matched healthy controls using the Low Input Quick Amp WT Labeling Kit (Agilent Technologies, USA). The tagged cRNAs were purified using the RNeasy Mini Kit (Qiagen, USA). A gene expression hybridization kit was used to hybridize 1.65 μg of Cy3-labeled cRNA on each slide for 17 hours while an Agilent microarray scanner was operating at its default settings. The data was extracted using Agilent Technologies’ Feature Extraction program 10.7. The Quantile technique and the R limma package were used to standardize the raw data. 31,977 lncRNAs in all were examined. Differentially expressed lncRNAs were retrieved based on count values using the edgeR package. Statistical analysis and generation of heatmaps and volcano plots for the expressed lncRNAs were conducted in either R or Perl environments.

### Cell lines

The colorectal cancer cell lines HT29 and SW480, as well as the human embryonic intestinal mucosa cell line CCC-HIE-2, were obtained from the American Type Culture Collection (ATCC, USA). According to the short tandem repeat (STR) analysis provided by the manufacturer, none of the cell lines used in this study were found in databases of commonly misidentified cell lines. While SW480 and CCC-HIE-2 cells were grown in Dulbecco’s Modified Eagle media (DMEM, Gibco, China), HT29 cells were grown in McCoy’s 5A media (Gibco).10% FBS (Gibco) and 1% Streptomycin/Penicillin (Gibco) were added to all culture media. The cells were incubated at 37 °C with 5% CO_2_ in a humidified atmosphere.

### Quantitative real-time polymerase chain reaction (RT-qPCR)

Total RNA from tissues or cells was purified using the TRIzol reagent (Invitrogen, USA) in accordance with the manufacturer’s instructions. RNA quality and concentration were assessed using a NanoDrop ND-2000 spectrophotometer (Thermo Fisher Scientific, MA, USA). One microgram of RNA was converted into cDNA using the TRUE script RT Kit (Proteinbio, Nanjing, China). Quantitative real-time PCR (RT-qPCR) was performed using a 7500 Real-Time PCR machine (Applied Biosystems, USA) and two Universal SYBR Green qPCR Supermixes (Proteinbio, Nanjing, China). The expression of B-actin acted as an internal regulator. All primers were provided by Sangon Biotech (Shanghai, China), and the sequences are in Supplementary Table S2.

### Cell transfection

Corues Biotechnology (Nanjing, China) provided us with human overexpression plasmids and lentiviral sh-LINC00183 so that we could create stable overexpression and knockdown CRC cell lines (Supplementary Table S4). The corresponding sequences for the specific small interfering RNAs (siRNAs) that GeneChem (Shanghai, China) chemically produced are given in Supplementary Table S1. Following the manufacturer’s instructions, transfections were carried out using Thermo Fisher Scientific’s Lipofectamine 3000TM reagent. Puromycin was utilized in the selection of stable cell lines.

### Western blotting

To extract the total protein from CRC cells, RIPA lysis buffer was used. An assay kit for BCA proteins (Leagene Biotechnology, Beijing, China) was used to measure the amount of protein. Cell lysates were separated using SDS-PAGE and then transferred onto PVDF membranes. Primary antibodies (mentioned in Supplementary Table S3) were added to the membranes at 4 °C for around 12 hours after they had been blocked with 5% skim milk for an hour at room temperature. After three TBST buffer washes, the membranes were incubated for two hours at room temperature with HRP-conjugated secondary antibodies. In the end, bands were observed using an ECL detection kit (Millipore, MA, USA) and a BioSpectrum 600 imaging system (Thermo Fisher Scientific).

### Immunoprecipitation

To extract the whole protein for immunoprecipitation assays, CRC cells were lysed in NP-40 lysis buffer (Beyotime, Shanghai, China) after being washed with cold PBS. Following the preclearing process using Protein A/G Plus-Agarose (SC-2003, Santa Cruz Biotechnology, CA, USA), certain antibodies were used to immunoprecipitate the lysates. As a non-specific control, purified immunoglobulin G (IgG) from the same host species (Cat. No. 12370, Sigma-Aldrich, MO, USA) was employed. To collect immune complexes, Protein A/G Plus-Agarose was used. Immunoprecipitated proteins were mixed with SDS-PAGE loading buffer and boiled for elution before being analyzed by Western blotting. Supplementary Table S3 contains a list of the antibodies utilized in this investigation.

### RNA pull-down assay

T7 RNA polymerase (Promega, WI, USA) was used to transcribe the full-length LINC00183 and its truncated segments from expression vectors in vitro. Following in vitro transcription, a Biotin RNA Labeling Mix (Roche, Basel, Switzerland) was used to biotinylate LINC00183 and its truncated fragments. Pierce’s Magnetic RNA-Protein Pull-Down Kit (Thermo Fisher Scientific) was used for pull-down tests. Streptavidin-conjugated magnetic beads were treated with biotin-labeled RNA for one hour at room temperature. At each stage, RNase inhibitors and protease/phosphatase inhibitor mixtures were added to freshly made whole-cell lysates. The lysates and bead-RNA complexes were incubated for six hours at 4 °C. After removing any loose proteins with a thorough cleaning, the RNA-protein complexes were boiled in SDS buffer. Silver staining was subsequently used to visualize the eluted proteins, and they were examined by western blotting and mass spectrometry.

### RNA immunoprecipitation (RIP) assay

RIP studies were performed using the Magna RNA-Binding Protein Immunoprecipitation Kit (Millipore, MA, USA) in accordance with the manufacturer’s instructions. RT-qPCR was used to verify immunoprecipitated RNA from HT29 and SW480 cells.

### CUT&Tag assay

As instructed by the manufacturer, the Cleavage Under Targets and Tagmentation (CUT&Tag) test was performed using the Hyperactive In situ ChIP Library Prep Kit for Illumina (Nanjing Vazyme, China). The resuspended cells were mixed with pre-treated concanavalin A-coated magnetic beads (ConA beads) and left to rest at room temperature to promote cell binding. The cell membranes were permeabilized with the nonionic detergent digitonin.ConA-bound cells were next treated with pA-Tn5 transposase, an appropriate secondary antibody, and anti-L-lactyl-histone H3 (Lys18) (H3K18la) antibody (Post-translational modifications [PTM]-1427RM, PTM BIO, Hangzhou, China). The cleaved DNA fragments were then ligated to P5 and P7 adaptors by the transposase and amplified by PCR using P5 and P7 primers. The purified PCR products were analyzed using an Agilent 2100 Bioanalyzer (Agilent Technologies). The libraries were then sequenced using an Illumina NovaSeq6000 platform (Illumina, Inc., USA), producing paired-end reads of 150 bp for further examination. The findings are displayed in Supplementary Files 5 (rep1) and 6 (rep2).

### Colony formation assay

Trypsinization, counting, and seeding of HT29 and SW480 cells at a density of 500 cells per well in 6-well plates were performed. For 14 days, the cells were cultivated at 37 °C in an environment containing 5% CO₂, with medium changes taking place every three days. After incubation, cells were rinsed, stained with 0.5% crystal violet, and fixed with 4% paraformaldehyde. To assess colony-forming efficiency, colonies with more than 50 cells were counted under a microscope.

### Cell counting kit-8 (CCK-8) proliferation assay

A density of 5000 cells per well was used to seed transfected cells in 96-well plates. Following 0, 24, 48, and 72 hours of incubation, 100 μL of culture media and 10 μL of CCK-8 reagent (KeyGEN, Nanjing, China) were added to each well. The wells were then incubated for two hours at 37 °C. At 450 nm, optical density (OD) was determined with a Thermo Fisher Scientific microplate reader. Each experiment was repeated three times.

### Ethynyl-20-deoxyuridine (EdU) assay

To further evaluate cell proliferation, the 5-ethynyl-2’-deoxyuridine (EdU) incorporation assay was employed. After being treated with 10 μM EdU, HT29 and SW480 cells were incubated for two hours at 37 °C. The cells were then permeabilized, fixed with 4% paraformaldehyde, and exposed to an azide-fluorochrome conjugate click chemical process. Nuclear staining was performed using DAPI. By using fluorescence microscopy to visualize and measure EdU-positive cells, the rate of proliferation was ascertained.

### Cell cycle analysis

Following a PBS wash, HT29 and SW480 cells were incubated for 30 minutes in a staining solution containing 100 μg/ml RNase A and 500 μg/ml propidium iodide (PI). This made it possible for PI to intercalate with the DNA and analyze the amount of DNA present in cells. Following incubation, the distribution of the cell cycle and DNA content were quantitatively evaluated by flow cytometry.

### Transwell assay

HT29 and SW480 cells were seeded in the upper chambers of Transwell inserts (Millicell; 8-µm pore size) covered with Matrigel after 500 µl of complete medium had been added to the lower wells. Following a 72-hour incubation period at 37 °C, non-migrating cells in the top chambers were gently removed with a cotton swab. Cells that migrated to the lower chambers were fixed for 10 minutes and then stained with 1% crystal violet in 2% ethanol for 15 minutes in order to measure cell migration.

### Wound-healing assay

After HT29 and SW480 cells were grown to around 90% confluence, a sterile pipette tip was used to make a linear scratch across the cell monolayer. The scratch was photographed under a microscope both right away (0 hours) and 48 hours later. ImageJ software (version 1.50i) was used to measure the average wound distance in order to evaluate cell migration and wound closure.

### Assays for glucose absorption, lactate generation, and ATP synthesis

CRC cells in 96-well plates were examined using a colorimetric L-lactate assay kit and a colorimetric glucose uptake assay kit, both from AAT Bioquest, CA, USA, in accordance with the manufacturer’s instructions. For the purpose of measuring ATP, cells grown in 6-well plates were lysed on ice using 200 μL of lysis solution per well, and then centrifuged at 4 °C. The Enhanced ATP Assay Kit from Beyotime Biotechnology, Shanghai, China, was used to measure the amount of ATP in the supernatant in accordance with the suggested technique. The results were then modified according to the number of cells. Each assay was independently executed five times.

### Glycolytic rate assay

Using Seahorse XF technology on a Seahorse XF-96 extracellular flux analyzer (Agilent, USA), the amount of glycolysis in CRC cells was measured. In short, each experimental group’s cells were cultured in triplicate for one hour in assay media without CO2, and then they were calibrated. Following successive injections of 2-DG (Sigma), oligomycin, and glucose, each group’s extracellular acidification rate (ECAR) was assessed. Data were normalized to protein content.

### Immunohistochemistry (IHC)

Serial sections embedded in paraffin, each 4 µm thick, were deparaffinized and rehydrated. Ten milliliters of Tris with one milliliter of EDTA (pH 9.0) were used to perform antigen retrieval in a pressure cooker for five minutes. The sections were immunodetected the next day after being treated with antibodies at 4 °C.

### Patient-derived organoid (PDO) culture model

Surgical CRC specimens were placed within sterile 10-cm dishes, rinsed in tissue dissociation medium (Hangzhou AimingMed Technologies Co., China), and finely minced into small fragments. Following the transfer of tissue pieces to a 15 mL conical tube, more tissue dissociation medium was added. The tube was incubated at 37 °C while being stirred. Under a microscope, the digestion process was continuously observed, and it was stopped when the majority of the cell clusters had a diameter of less than 200 μm. A 100-μm cell strainer was then used to filter the mixture after it had been resuspended in DPBS. Centrifugation at 300 *g* for 5 minutes was used to gather the filtered cells, which were then resuspended in Matrigel and dispensed as 50 μL droplets into the middle of each well of a 24-well plate. After five minutes of incubation to let the Matrigel to set, the plate was inverted for a further twenty-five minutes to allow the Matrigel dome to form. Then, without disturbing the domes, 600 μL of pre-warmed organoid complete medium (AimingMed Technologies Co.) was gently added to each well. The entire medium was replaced every two to three days while the plate was kept at 37 °C in an incubator with 5% CO₂. At room temperature, the spent medium was taken out and replaced with brand-new, full medium.

### Animal experiments

WeiTongHuaLi (China) provided the four-week-old male BALB/c athymic nude mice, which were housed in compliance with Nanjing Medical University’s Institutional Animal Care and Use Committee guidelines (Approval No. IACUC-2211031). Two × 10⁶ LINC00183-knockdown, overexpression, or control HT29 cells suspended in 100 μL of cold PBS were subcutaneously injected into the dorsal flanks of the mice. At the conclusion of the trial, the tumors were removed, weighed, and photographed, and the mice were kindly put to sleep.

Luciferase-labeled HT29 cells (1 × 10⁶ cells in 100 μL of PBS) were injected into the tail vein of BALB/c athymic nude mice in order to assess the in vivo lung metastasis of CRC cells. In order to identify metastatic lung lesions, the mice were given D-luciferin sodium salt (150 mg/mL) after five weeks of anesthesia with 2% isoflurane.

Additionally, we created a model of liver metastases from colorectal cancer via splenic injection. A single-cell suspension of 1 × 10⁶ luciferase-expressing HT29 cells was prepared using 200 μL of PBS. Male naked mice (6–8 weeks old, 16–22 g) had a 2–3 cm subcostal incision done on their left side following anesthesia. The spleen was separated into two hemispans and sutured with 4–0 after it had been exposed. A 27G syringe was used to fill one hemispan with 100 μL of the cell suspension. After roughly 10 minutes to allow for liver metastases, the injected hemispan was taken out and the remaining hemispan was reinserted. The incision was then sealed. Bioluminescence imaging was performed on days 0, 6, 10, and 14 post-injection to monitor the development of tumor metastases following a three-minute luciferase substrate administration.

An IVIS Spectrum imaging system (Caliper Life Sciences, Hopkinton, MA, USA) was used to record bioluminescence. Following imaging, the mice were put to sleep, and their lungs and livers were taken out for H&E staining. Every technique complied with the guidelines established by the Nanjing Medical University Committee for the Use and Care of Laboratory Animals and was ethically approved.

During the allocation process, no animals were deliberately included or excluded. Each group was randomly assigned five male BALB/c mice.

## Results

### Exosomes derived from platelets of CRC patients promote malignant biological behavior of CRC cells in vitro

**Fig. 1:**
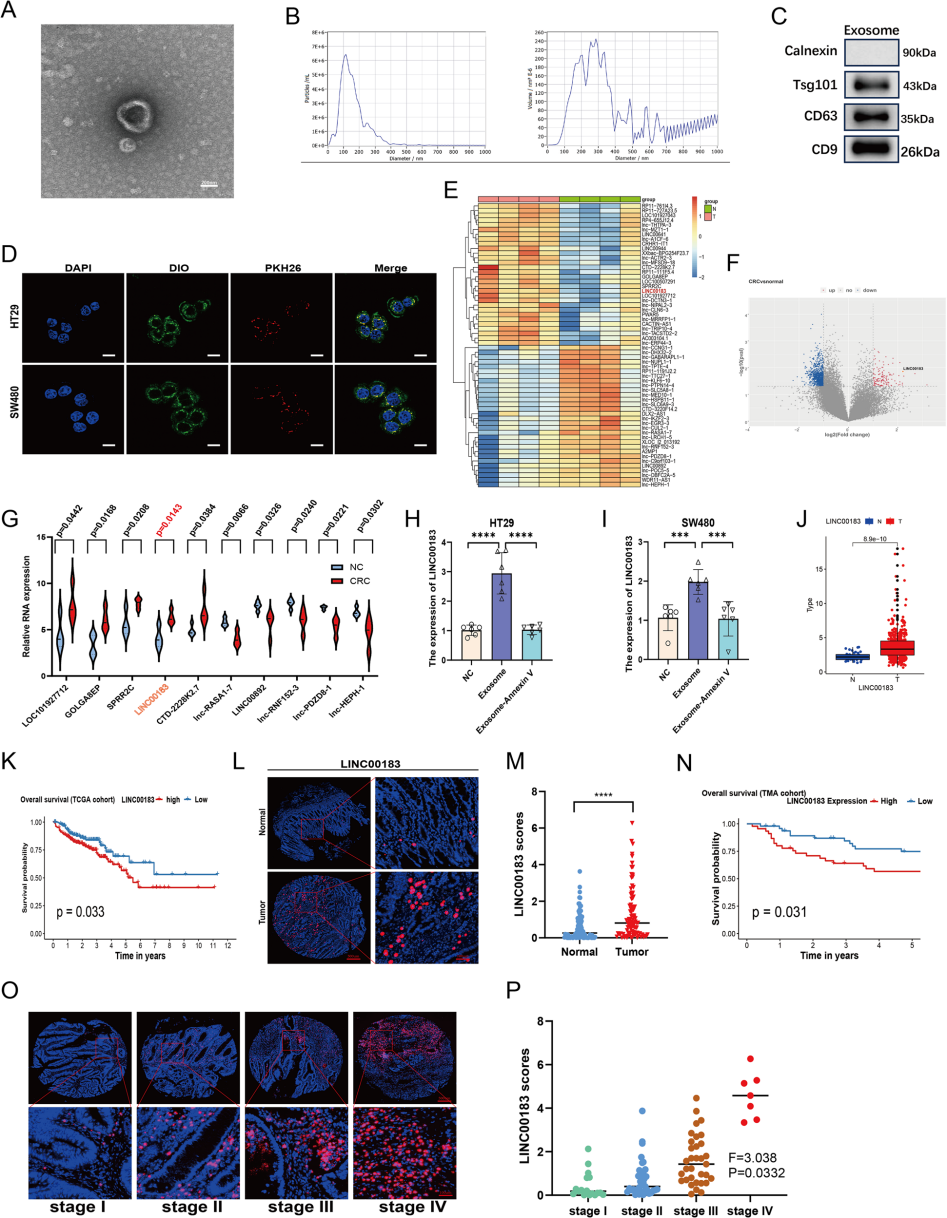
Platelet-derived exosomal LINC00183 is upregulated in CRC and exhibits clinical significance. **A** TEM analysis of the morphology of PLT-Exos. Scale bar, 200 nm. **B** NTA analysis of PLT-Exos. **C** Western blot analysis of exosomal markers (CD9, CD63, and TSG101) and Calnexin (negative control) in PLT-Exos. **D** Confocal microscopy images of PKH26-labeled exosomes internalized by HT29 and SW480 cells. Scale bar, 10 μm. **E** Heatmap illustrating differential lncRNA expression between PLT-Exos derived from CRC patients and healthy individuals. **F** Volcano plot indicating significant differences in lncRNA expression between PLT-Exos derived from CRC patients and healthy individuals. **G** PLT-Exos from CRC patients and healthy individuals showed different levels of expression for the top five lncRNAs that were elevated and downregulated. **H**, **I** HT29 and SW480 treated with or without Annexin V were incubated with CRC/PLT-Exos and cellular LINC00183 expression was measured by RT-qPCR. **J** LINC00183 expression between CRC tissues and normal colon samples in the TCGA database. **K** Overall survival (OS) in TCGA-CRC patients was analyzed using the Kaplan-Meier method based on the expression of LINC00183 (*P* = 0.033). **L**, **M** FISH-based analysis of LINC00183 expression in CRC tissues and matched normal tissues. Scale bar, 200 μm. **N** Prognostic analysis of LINC00183 in 93 CRC cases from our center. **O**, **P** FISH-based analysis of LINC00183 expression in CRC samples with different TNM stage. Scale bar, 200 μm. *P < 0.05; **P < 0.01; ***P < 0.001; ns, no significance.

PLT-Exos purified from peripheral blood of CRC patients (CRC/PLT-Exos) were characterized using TEM and NTA as spherical particles with a characteristic diameter of approximately 100 nm (Fig. 1A and B). Western blotting results demonstrated prominent bands for the exosomal markers TSG101, CD63, and CD9, while the exosomal negative marker Calnexin was absent (Fig. 1C). Subsequently, the exosomes were added to human HT29 and SW480 CRC cells to evaluate uptake and effect on proliferation and migration. Confocal imaging revealed the accumulation of exosomes in the cytoplasm, indicating exosome internalization (Fig. 1D).

Subsequently, CCK-8, EdU, Transwell, and cell scratch assays demonstrated that, compared to the blank control, the addition of CRC/PLT-Exos significantly enhanced the proliferation and migration capabilities of HT29 and SW480 cells (Supplementary Fig. S1A-H).

### RNA-seq-based identification of lncRNAs in PLT-Exos

LncRNAs are stably and abundantly present in exosomes, and may act as key regulatory factors in cancer [16]. We thus evaluated whether CRC is associated with differential lncRNA expression in PLT-Exos using RNA sequencing (RNA-seq). A total of 1097 differentially expressed lncRNAs (FC ≥ 1.5, *P* < 0.05) were identified between PLT-Exos derived from CRC patients and from healthy individuals (Fig. 1E and F)(Supplementary File1). Among all the differentially regulated lncRNAs in CRC/PLT-Exos, LINC00183 showed marked overexpression and exhibited the lowest *p*-value (Fig. 1G). When HT29 and SW480 cells were indirectly (using Transwell chambers) or directly co-cultured with CRC/PLT-Exos, increased levels of LINC00183 were detected in both cell types by RT-qPCR (Supplementary Fig. S1I and J). In turn, upon treatment with Annexin V, which was reported to inhibit exosome internalization by blocking exposed phosphatidylserine at the exosomal surface [REF], cellular levels of LINC00183 were decreased (Fig. 1H and I). These observations indicate that LINC00183 is strongly upregulated in CRC/PLT-Exos and can be transferred to CRC cells.

### LINC00183 is highly expressed in CRC

According to an analysis of the Cancer Genome Atlas (TCGA) database, CRC had higher levels of LINC00183 expression than normal colon tissue (Fig. 1J), and that compared to patients with low LINC00183 expression, those with high LINC00183 expression had noticeably worse prognoses (Fig. 1K). LINC00183 expression was next assessed by FISH in a tissue microarray comprising 96 pairs of tumor and matched normal tissues from CRC patients. The findings showed that tumor tissues had substantially higher levels of LINC00183 than normal tissues (Fig. 1L and M). Furthermore, elevated LINC00183 expression was positively correlated with poor prognosis (Fig. 1N) and may be associated with advanced pathological stages (Fig. 1O, P). These data further confirm that LINC00183 is significantly upregulated in CRC tissue and may serve as a potential lncRNA biomarker for early diagnosis.

### LINC00183 facilitates CRC cell growth in vitro and in vivo

To explore whether LINC00183 influences the progression of CRC, we constructed three siRNAs targeting LINC00183, along with LINC00183 overexpression plasmids. The efficacy of the siRNAs and overexpression plasmids was validated through RT-qPCR (Supplementary Fig. S2A, B). We performed gain-of-function and loss-of-function studies on the three siRNAs and chose si-LINC00183#1 and si-LINC00183#2, which showed more noticeable knockdown effects in HT29 and SW480 cells.

**Fig. 2:**
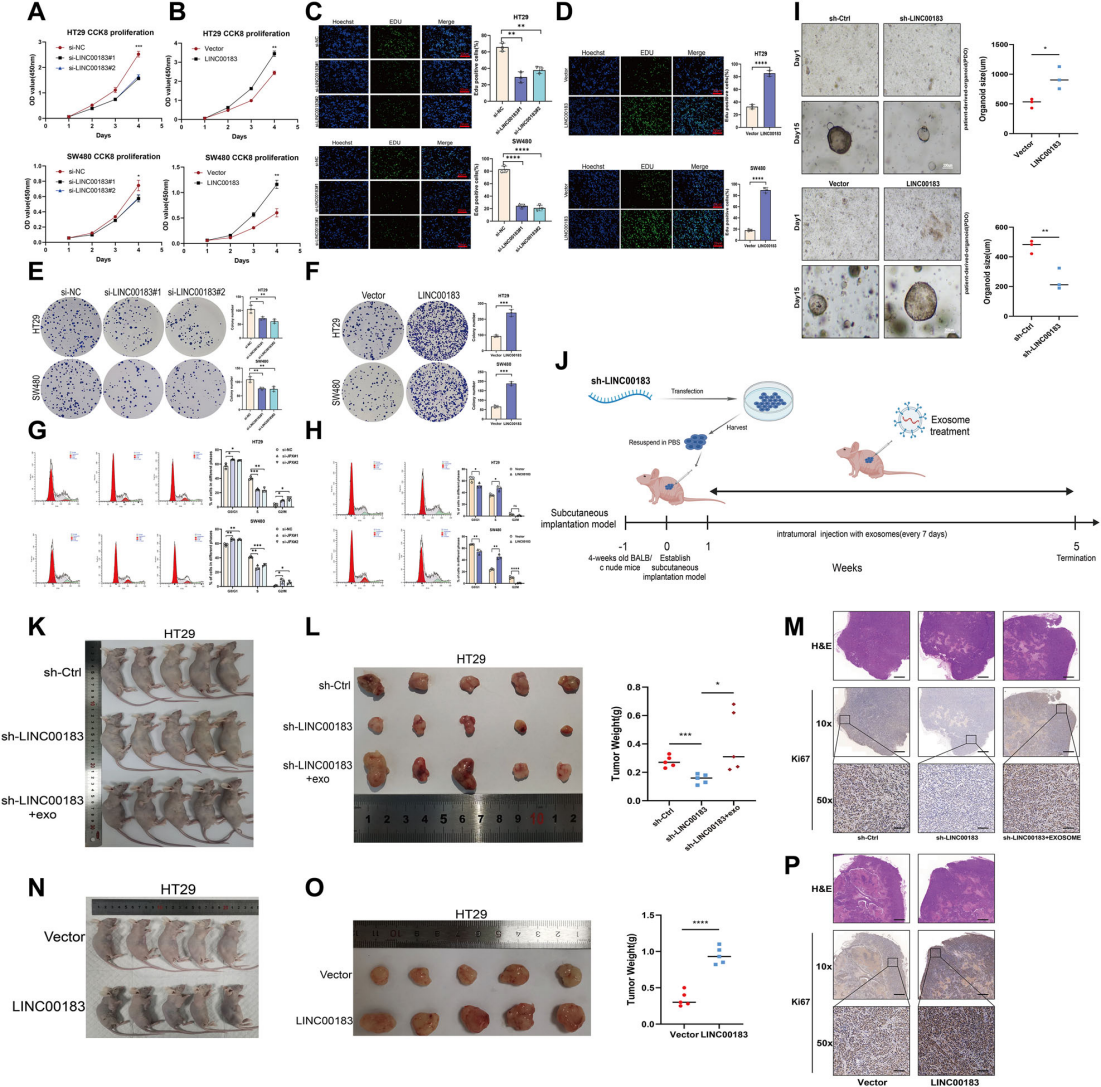
LINC00183 facilitates CRC growth in vitro and in vivo. **A–D** Results of CCK-8 and EdU assays illustrating the effect of LINC00183 overexpression and silencing on the proliferative capacity of CRC cell lines. Scale bar = 50 μm. **E**, **F** Effect of LINC00183 silencing and overexpression on the clonogenic ability of CRC cells. **G**, **H** Flow cytometry-based analysis of cell cycle staging (PI staining) on HT29 and SW480 cells following LINC00183 knockdown and overexpression. **I** Representative bright-field images of 3D organoids derived from CRC patients following LINC00183 overexpression, knockdown, or control treatment. **J** Diagram showing the CRC xenograft model in naked mice. **K**, **L**, **N**, and **O** Tumor weight measurements and representative pictures of mice with subcutaneous colorectal cancer tumors. The mean ± SEM of five biologically distinct samples is presented in the data. **M** and **P** Ki-67 IHC and representative H&E staining of removed CRC xenograft sections.

CCK-8, EdU, and clonogenic assays revealed that transfection of si-LINC00183#1 or si-LINC00183#2 significantly diminished the proliferative capacity of HT29 and SW480 cells (Fig. 2A, C, and E). Conversely, the overexpression of LINC00183 markedly enhanced cell proliferation (Fig. 2B, D, and F). Cell cycle analyses indicated that upregulation of LINC00183 led to G0/G1 cell cycle arrest, while its downregulation had the opposite effect (Fig. 2G, H). Furthermore, we generated PDOs from CRC tissues and found that LINC00183 knockdown and overexpression inhibited and promoted, respectively, PDO formation (Fig. 2I). These results demonstrated that LINC00183 enhances the proliferation of CRC cells.

Next, we investigated the impact of LINC00183 on CRC growth in vivo using LINC00183 knockdown and LINC00183-overexpressing CRC HT29 cells to establish a subcutaneous xenograft model in BALB/c nude mice (Fig. 2J). Tumor weight measurements and Ki-67 IHC on excised tumor sections indicated that LINC00183 overexpression promoted tumor growth (Fig. 2N–P), whereas LINC00183 silencing inhibited tumor progression (Fig. 2K–M). Notably, intratumoral injection of CRC/PLT-Exos was able to reverse the inhibitory effect of LINC00183 silencing on CRC growth (Fig. 2K–M). These findings showed that LINC00183 promotes the growth of colorectal cancer cells both in vitro and in vivo.

### LINC00183 promotes CRC metastasis

**Fig. 3:**
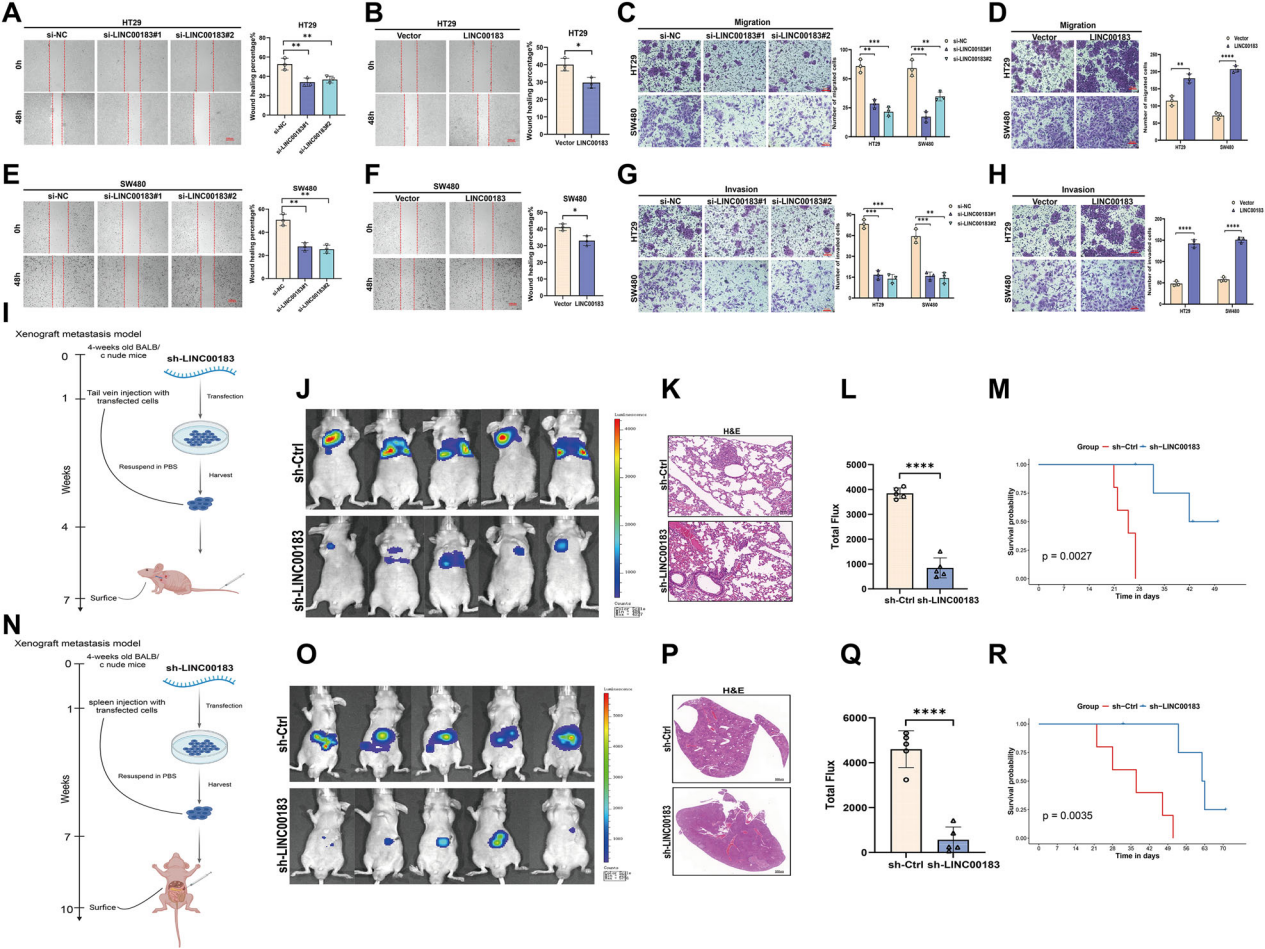
LINC00183 facilitates CRC metastasis in vitro and in vivo. **A**–**H** Results of Transwell and wound-healing assays examining the effect of LINC00183 on CRC cell migration and invasion. Scale bars, 50 μm. **I** Schematic representation of the tail vein injection model for lung metastasis. **J**–**M** Shown are representative images of bioluminescent detection of lung metastases (**J**), overall survival (OS) curves (**M**), and total photon flux measurements (**L**). H&E-stained serial lung tissue sections are shown in (**K**). Scale bars, 500 μm. **N**–**Q** Schematic of the spleen injection liver metastasis model. Shown are representative images of bioluminescent detection of liver metastases (**O**), OS curves (**R**), and total photon flux measurements (**Q**). H&E-stained serial liver tissue sections are shown in (**P**). Scale bars, 500 μm.

Wound-healing assays demonstrated that the transfection of si-LINC00183#1 or si-LINC00183#2 significantly reduced the migratory capacity of HT29 and SW480 cells (Fig. 3A, E), whereas LINC00183 overexpression markedly stimulated it (Fig. 3B, F). Likewise, on Transwell assays, the invasive potential of HT29 and SW480 cells was significantly reduced after LINC00183 knockdown (Fig. 3C, G) and stimulated instead following LINC00183 overexpression (Fig. 3D, H).

We created metastatic lung and liver colonization models in BALB/c nude mice by injecting luciferase-labeled HT29 cells that were stably transfected with sh-LINC00183-encoding lentiviruses via the spleen and tail vein, respectively, in order to determine whether LINC00183 increases the metastatic potential of CRC cells in vivo (Fig. 3I, N). The dissemination of HT29 cells to the lung and liver was assessed by in vivo bioluminescence (Fig. 3J, O), which indicated that LINC00183 downregulation weakened the ability of CRC cells to form lung and liver metastases (Fig. 3L, Q). The mice were put to sleep after ten weeks, and the liver and lungs were removed for H&E staining. The findings verified that after LINC00183 silencing, there were fewer metastatic nodules (Fig. 3K, P). Moreover, survival analysis demonstrated that silencing LINC00183 led to improved survival prognosis in the mice (Fig. 3M, R). These results demonstrated that LINC00183 facilitates CRC cell metastasis in vitro and in vivo.

### LINC00183 promotes aerobic glycolysis in CRC

**Fig. 4:**
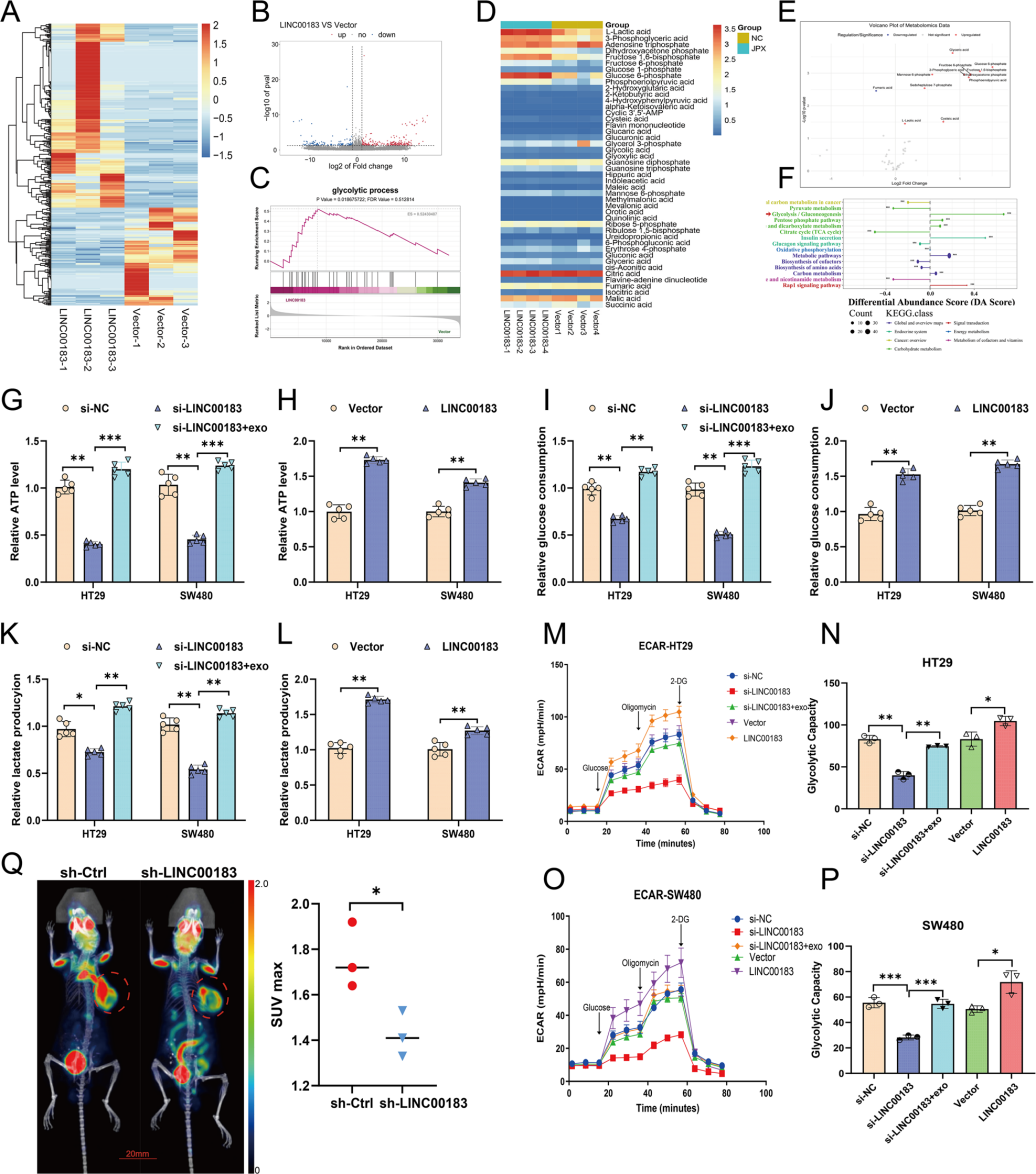
LINC00183 promotes aerobic glycolysis in CRC. **A** Heatmap of all differentially expressed genes in HT29 cells transfected with LINC00183 overexpression or control plasmids. **B** Volcano plot of transcriptome sequencing data. **C** GSEA results showing DEG enrichment in the glycolysis pathway. **D** Heatmap of all differentially expressed metabolites in HT29 cells transfected with LINC00183 overexpression or control plasmids. **E** Volcano plot of central carbon metabolism from metabolomics sequencing data. **F** GSEA of central carbon metabolism metabolites. **G**–**L** Analysis of glucose uptake, ATP production, and lactate generation in cultured HT29 CRC cells. **M–P** ECAR measurements in cultured HT29 CRC cells. **Q** PET-CT analysis of glucose uptake in mice bearing subcutaneous CRC xenografts.

To elucidate the molecular mechanisms through which LINC00183 promotes CRC tumorigenesis and growth, we conducted RNA-seq on HT29 cells alternatively transfected with LINC00183 overexpression and control plasmids. According to our findings, there were 2030 genes (DEGs) that were differently expressed across the two groups (*P* < 0.05, |log FC |≥1)(Fig. 4B) (Supplementary File 2). The RNA heatmap indicated that LINC00183 overexpression is associated with the dysregulation of distinct subsets of transcripts (Fig. 4A). In turn, Gene Set Enrichment Analysis (GSEA) demonstrated that the DEGs are predominantly enriched in the glycolytic pathway (Fig. 4C) (Supplementary File 2).

Glycolytic metabolites such as glucose-6-phosphate (G6P), 3-phosphoglycerate (3-PGA), dihydroxyacetone phosphate (DHAP), fructose-1,6-bisphosphate (FBP), fructose-6-phosphate (F6P), phosphoenolpyruvate (PEP), and lactate were found to be elevated in response to LINC00183 overexpression, according to additional targeted metabolomic analysis using LC/mass spectrometry (MS) (Fig. 4D, E)(Supplementary File 3). Additionally, functional analysis of the metabolomic data revealed that glycolytic activity was most pronounced in the LINC00183 overexpression group (Fig. 4F)(Supplementary File 3). Collectively, these findings suggest that LINC00183 acts as a positive regulator of glycolysis in CRC cells.

We assessed the amount of lactate, ATP, and glucose uptake in cultivated colorectal cancer cells in order to test this theory. The findings showed that LINC00183 knockdown resulted in decreased lactate production, ATP synthesis, and glucose absorption. In contrast, glucose uptake, ATP levels, and lactate production were increased after co-culture with CRC/PLT-Exos (Fig. 4G–L). By assessing the ECAR of HT29 cells in vitro, we next determined the impact of LINC00183 silencing on the cells’ glycolytic rate. Notably, a significantly reduced ECAR was observed upon LINC00183 deletion, and this reduction was reversed by co-culture with CRC/PLT-Exos (Fig. 4M–P). Moreover, in vivo PET-CT imaging revealed that silencing LINC00183 markedly suppressed glucose metabolism in subcutaneous CRC xenografts in mice (Fig. 4Q). These results suggest that LINC00183 acts as a positive regulator of glycolysis in colorectal tumors.

### LINC00183 directly interacts with ENO1

**Fig. 5:**
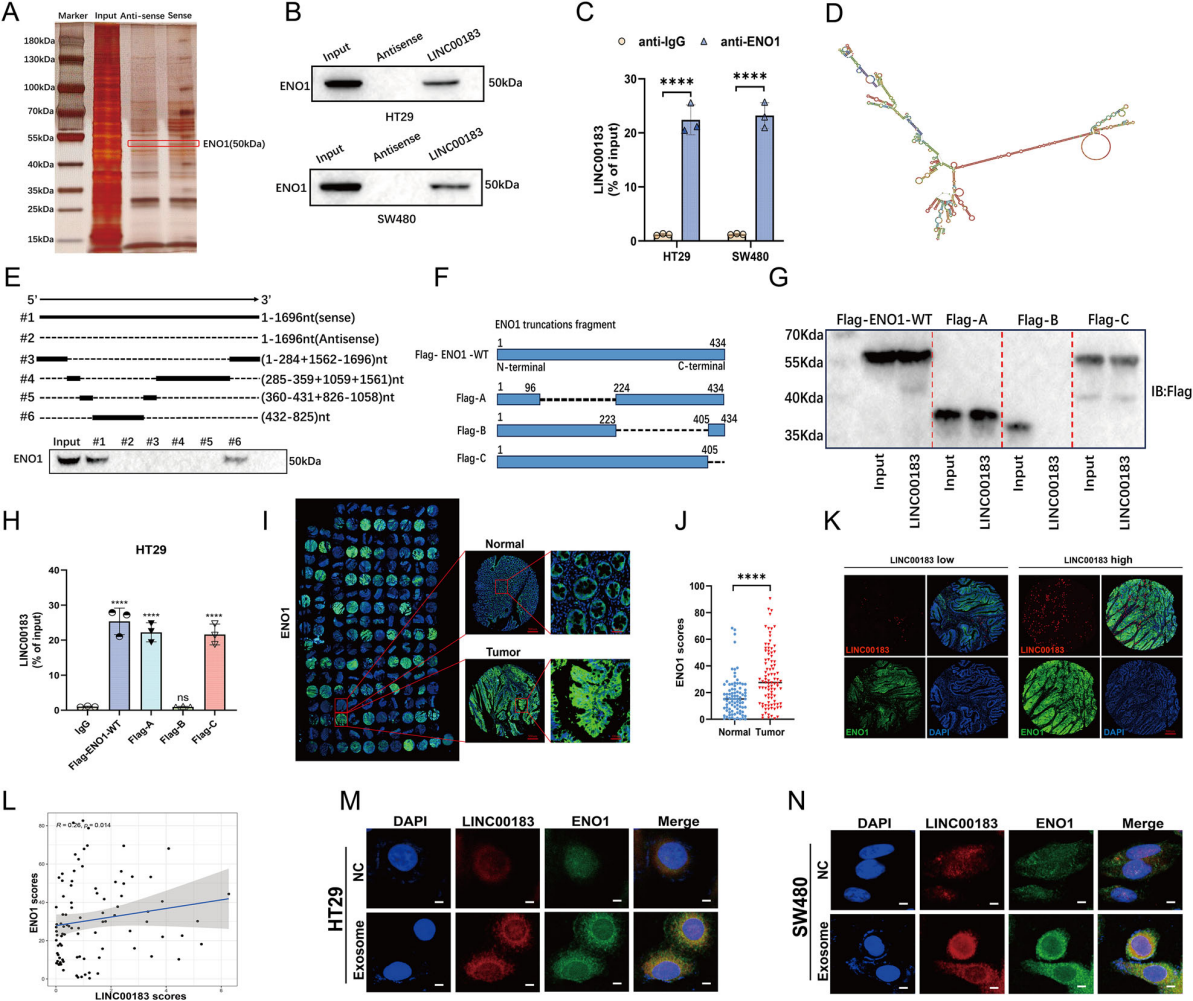
LINC00183 directly interacts with ENO1. **A** Representative results of SDS-PAGE and silver staining assays using pull-down fractions of LINC00183 sense and antisense probes and HT29 cell lysates. **B** ENO1 was found to be enriched in the fractions that co-eluted with LINC00183 in RNA pull-down assays, according to Western blot analysis. **C** Western blot analysis confirming that ENO1 was enriched in the fractions co-precipitated with LINC00183 in RIP assays. **D** Secondary structure of LINC00183, as predicted by RNAfold. **E** Diagrammatic illustration of the LINC00183 truncated mutant pieces (top panel). Fragment #6 of LINC00183 is necessary for its interaction with ENO1, according to Western blot analysis (bottom panel). **F** Diagrammatic representation of ENO1 plasmids that are either truncated mutant or FLAG-tagged wild-type (WT). **G** Western blot analysis revealed that deletion of the 224–404 aa region in ENO1 hindered the ability of LINC00183 to bind ENO1. **H** RIP assay results showing that deletion of aa 224–404 in the ENO1 protein prevents its binding to LINC00183. **I**, **J** IF analysis of ENO1 expression in CRC samples and matched normal colon tissue in tissue microarrays. Scale bar, 200 μm. **K** and **L** Correlation between LINC00183 and ENO1 expression. Data are derived from IF analysis of CRC tissue microarrays. **M** and **N** Verification of LINC00183 and ENO1 co-localization obtained through RNA-FISH and IF analyses in HT29 and SW480 cells. Scale bar, 20 μm.

The function of lncRNAs is closely related to their subcellular localization. According to fluorescence in situ hybridization (FISH) tests, LINC00183 is found in the cytoplasm and nucleus of colorectal cancer cells (Supplementary Fig. S2C). Since the interaction between lncRNAs and proteins was shown to influence cancer progression, we employed the biotin-RNA pull-down assay followed by MS analysis to identify potential LINC00183-binding proteins. In HT29 cells, approximately 235 potential interacting proteins were co-precipitated with LINC00183 (Supplementary File 4). Among these, alpha-enolase (Enolase 1; ENO1) was identified as a LINC00183 partner (Fig. 5A). ENO1 is a glycolytic enzyme that catalyzes the conversion of 2-phosphoglycerate to phosphoenolpyruvate in the glycolytic pathway [17] and is frequently overexpressed in tumor cells in association with cancer progression [18]. Subsequently, we validated the physical interaction between LINC00183 and ENO1 using biotin-RNA pull-down assays and RNA immunoprecipitation (RIP) experiments (Fig. 5B, C).

The secondary structure of LINC00183 was predicted using RNAfold software (http://rna.tbi.univie.ac.at/cgi-bin/RNAWebSuite/RNAfold.cgi) (Fig. 5D). To elucidate the binding region between LINC00183 and ENO1, we constructed two biotin-labeled full-length LINC00183 constructs along with four biotin-labeled LINC00183 fragments: #1: full-length LINC00183 sense strand; #2: full-length LINC00183 antisense strand; #3: exon (1-284 + 1562-1696 nt); #4: exon (285-359 + 1059 + 1561 nt); #5: exon (360-431 + 826-1058 nt); and #6: exon (432–825 nt)(Supplementary Table S5). These constructs were used to HT29 cell lysates in RNA pull-down assays. The LINC00183 area, which spans nt 432-825, is crucial for its interaction with ENO1, according to deletion mapping studies(Fig. 5E). The N-terminal domain (NTD), the cold shock domain (CSD), and the big C-terminal domain (CTD) are the three structural domains that make up ENO1 [17]. We cloned a number of shortened ENO1 proteins using a FLAG tag in order to determine the domains involved in the binding of ENO1 to LINC00183: FLAG-C (1-104 aa); FLAG-B (1-222 + 405-434 aa); and FLAG-A (1-96 + 224-434 aa)(Supplementary Table S6) (Fig. 5F). After that, we used full-length LINC00183 to conduct pull-down studies, which showed that LINC00183 could not capture the FLAG-C shortened fragment(Fig. 5G). Further evidence that the 224–404 amino acid region of ENO1 is necessary for its interaction with LINC00183 was provided by RIP studies, which likewise revealed no relationship between the FLAG-B shortened segment and LINC00183 (Fig. 5H). These results indicated that the 432–825 nt fragment of LINC00183 is responsible for binding to the 224–404 amino acid region of ENO1.

We next assessed the expression of LINC00183 and ENO1 protein in clinical CRC tissue microarrays. Results revealed significantly higher ENO1 expression in CRC tissues compared to adjacent normal colon (Fig. 5I, J). Furthermore, a significant positive correlation was observed between LINC00183 and ENO1 protein expression levels (Fig. 5K, L). In turn, co-localization experiments using FISH and immunofluorescence (IF) demonstrated that LINC00183 and ENO1 are co-localized in the nucleus and cytoplasm of HT29 cells. Interestingly, cytoplasmic co-localization was promoted by co-culturing cells with CRC/PLT-Exos (Fig. 5M, N).

### LINC00183 stabilizes ENO1 by attenuating its ubiquitin-proteasome-mediated degradation

**Fig. 6:**
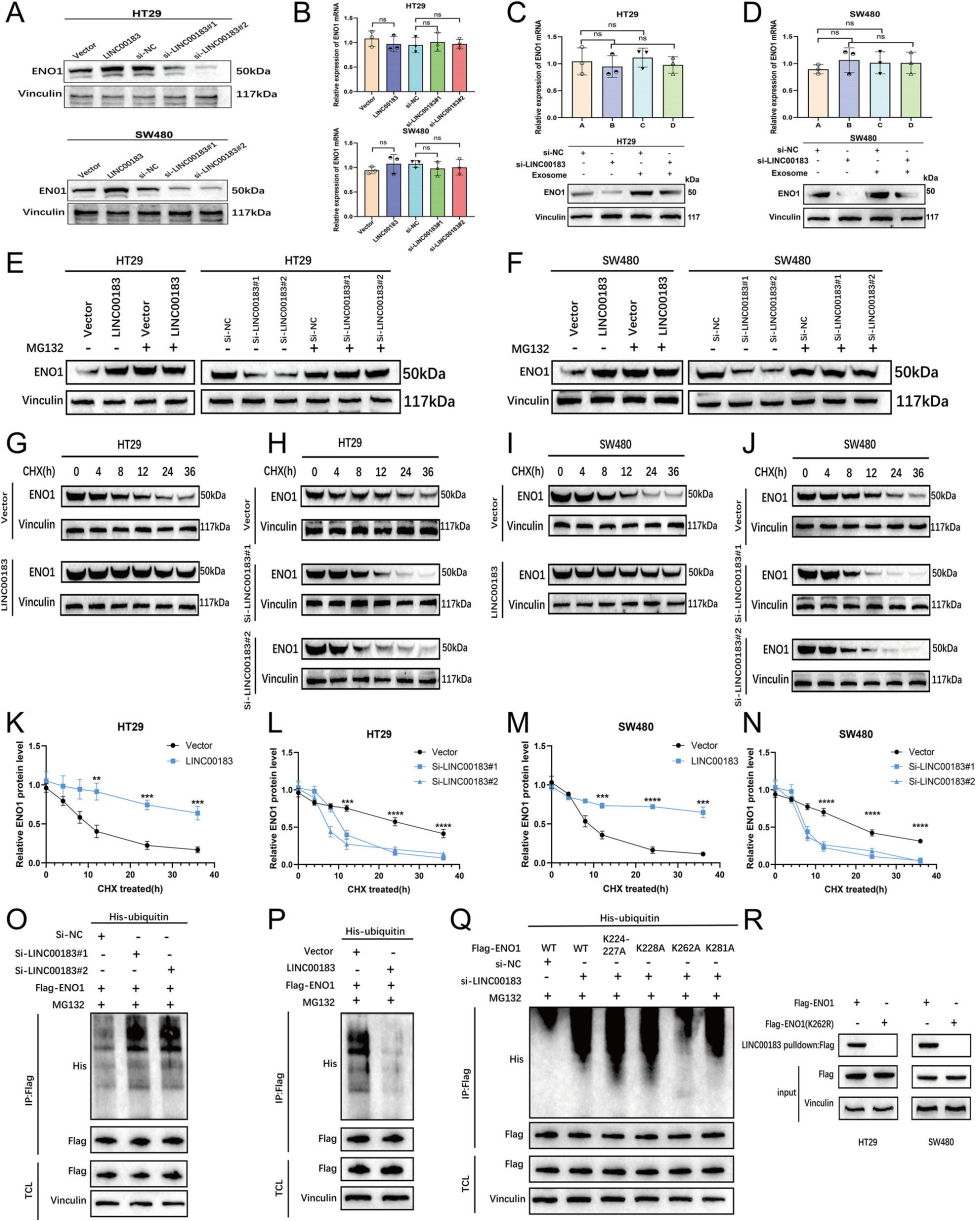
LINC00183 stabilizes ENO1 by inhibiting its degradation through the ubiquitin-proteasome system. **A**, **B** HT29 and SW480 cells’ relative mRNA and protein ENO1 expression after LINC00183 overexpression or knockdown were examined using RT-qPCR and western blot techniques. **C**, **D** RT-qPCR and western blot analyses of ENO1 expression in HT29 and SW480 cells co-cultured with CRC/PLT-Exos. **E**, **F** ENO1 expression in CRC cells treated with or without MG132 (10 μmol/L for 10 hours) was examined using Western blot. **G**–**J** Western blot analysis ENO1 expression after LINC00183 overexpression or silencing in CHX-treated CRC cells. **K**–**N** Time course of changes in relative ENO1 protein levels. **O**, **P** Co-IP and western blot analyses examining ENO1 ubiquitination in HT29 cells co-transfected with His-tagged ubiquitin, FLAG-tagged ENO1, and either si-LINC00183, LINC00183, or the corresponding controls. TCL: total cell lysate. **Q** Co-IP and western blot analyses of ENO1 ubiquitination levels in HT29 cells transfected with plasmids expressing FLAG-tagged WT or mutant (K224-227A, K228A, K262A, and K281A) ENO1 protein along with His-tagged ubiquitin plasmids and LINC00183-targeted siRNA or control siRNA. **R** Plasmids expressing FLAG-tagged ENO1 or FLAG-tagged mutant ENO1 (K262A) were used to transfect HT29 and SW480 cells. The LINC00183 probe was then used in a lncRNA pull-down assay, and a particular anti-FLAG antibody was used for western blot analysis.

According to earlier research, lncRNAs control the stability of their binding proteins or act as molecular scaffolding for structural and functional events, among other ways [19]. Therefore, we first examined whether LINC00183 affects the expression of ENO1. RT-qPCR and western blot analyses indicated that after LINC00183 knockdown or overexpression, ENO1 mRNA expression remained unchanged, while ENO1 protein levels were, respectively, decreased and increased in HT29 and SW480 cells (Fig. 6A and B). Furthermore, co-culturing with CRC/PLT-Exos increased ENO1 protein content, but not its mRNA levels, in CRC cells (Fig. 6C, D). These data suggest that in CRC patients, post-transcriptional ENO1 expression in CRC cells is upregulated by the transfer of platelet-derived exosomal LINC00183.

Ubiquitination is a well-known post-translational modification in which proteins are marked for degradation by ubiquitin. Tumorigenesis is one of the many physiological and pathological processes in which this phenomenon is crucial [20]. Thus, we explored whether LINC00183 sustains ENO1 levels by preventing its degradation through the ubiquitin-proteasome pathway. Compared to DMSO-treated control CRC cells, exposure to the ubiquitin-proteasome inhibitor MG132 mitigated ENO1 degradation induced by LINC00183 knockdown (Fig. 6E, F). Additionally, protein degradation assays were performed using cycloheximide (CHX), a protein synthesis inhibitor. We found that ENO1 degradation was inhibited and promoted, respectively, by LINC00183 overexpression and knockdown (Fig. 6G–N). These results indicate that LINC00183 may act as a stabilizing factor for ENO1.

Then, by co-transfecting HT29 and SW480 cells with FLAG-tagged ENO1 and His-tagged ubiquitin, as well as either si-LINC00183, LINC00183, or the corresponding controls, we evaluated the degree of ubiquitination of ENO1. Subsequently, the HT29 cells were treated (10 hours) with MG132 (10 μmol/L) or DMSO (control). Co-immunoprecipitation (co-IP) assays performed using anti-FLAG antibodies and western blot assays conducted with anti-His antibodies demonstrated that silencing LINC00183 increased the ubiquitination level of ENO1, while overexpression of LINC00183 had the opposite effect (Fig. 6O, P and Supplementary Fig. S2D, E). Thus, we conclude that LINC00183 enhances ENO1 stability by binding to ENO1 and inhibiting its ubiquitin-proteasome-mediated degradation.

As previously stated, mapping tests demonstrated that the 224–404 aa region of ENO1 interacts with the 432–825 nt segment of LINC00183. Next, we looked for evidence that the lysine residues in this peptidic area contribute to ENO1 ubiquitination. CPLM (http://cplm.biocuckoo.org/) and PhosphoSite (https://www.phosphosite.org/) are two online tools that we used to identify four possible lysine residues (K224-227, K228, K262, and K281) within the LINC00183-binding region of ENO1 that might be involved in ENO1 ubiquitination. Subsequently, we constructed wild-type (WT) and mutant plasmids of ENO1 for ubiquitination assays. The results showed that the K262 (K262A) mutation reduced ENO1 ubiquitination levels, while mutations at K224-227 (K224-227A), K228 (K228A), and K281 (K281A) had no effect (Fig. 6Q and Supplementary Fig. S2F). Finally, RNA pull-down and western blot assays demonstrated that the K262 mutation disrupted the interaction between LINC00183 and ENO1 (Fig. 6R), while mutations at the other three sites were without effect (Supplementary Fig. S2D–F). We deduce that LINC00183 binds to the 224–404 aa stretch of ENO1 and obscures the K262 residue, preventing ubiquitin-proteasome-mediated degradation of ENO1.

### LINC00183 promotes histone lactylation

**Fig. 7:**
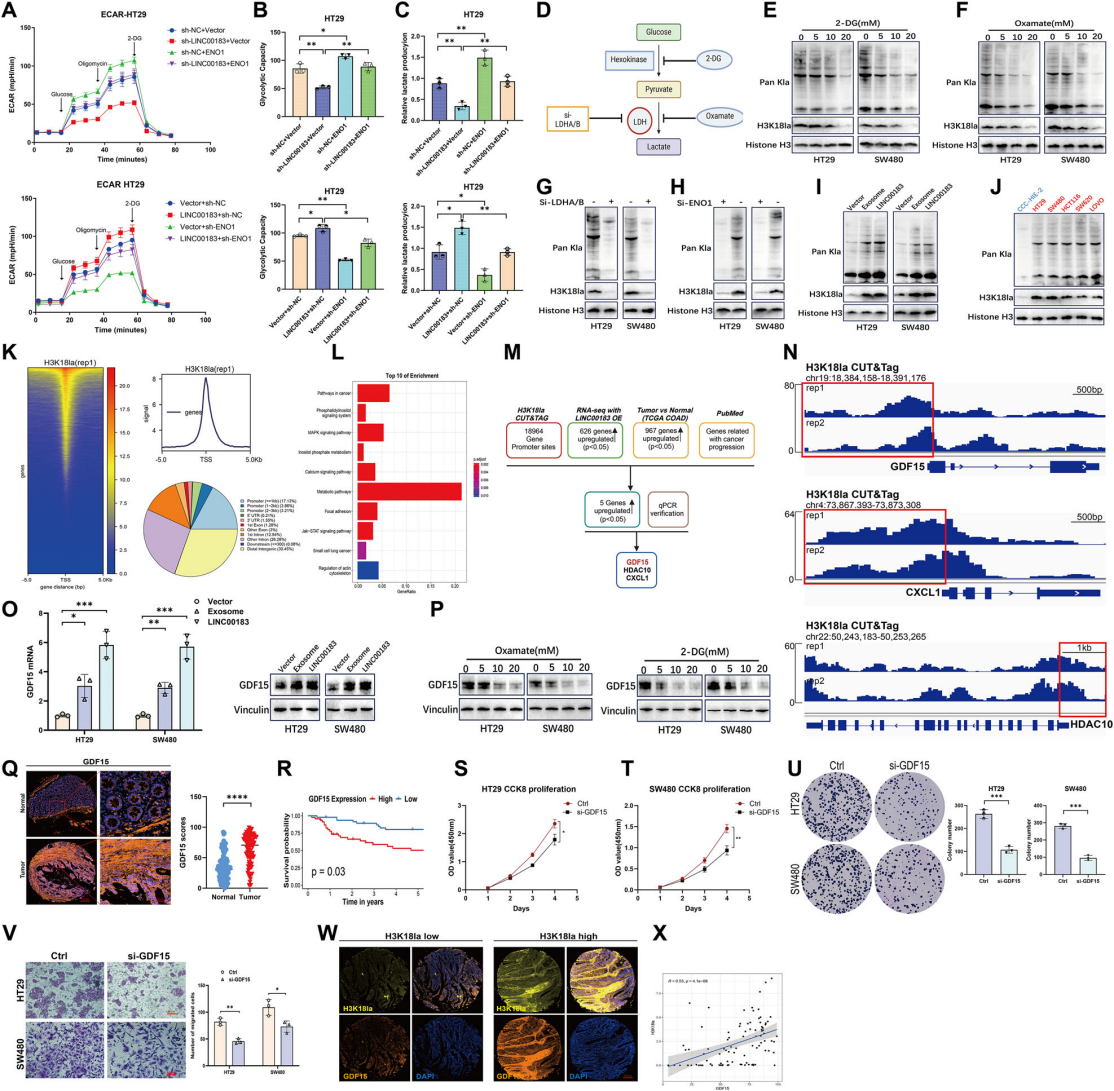
LINC00183-mediated H3K18 lactylation promotes CRC progression by stimulating GDF15 expression. **A**, **B** ECAR measurements in HT29 cells following ENO1/LINC00183 knockdown/overexpression. **C** Lactate production assay results in HT29 cells following ENO1/LINC00183 knockdown/overexpression. **D** Schematic of glycolysis, highlighting the methods used in this study to inhibit lactate production and histone lactylation. **E** and **F** Western blot study of H3K18 lactylation and Pan Kla levels in CRC cells treated for 24 hours with varying doses of 2-DG or Oxamate. **G** The impact of LDH deficiency on Pan Kla and H3K18 lactylation was evaluated by Western blotting. **H** The impact of ENO1 deficiency on Pan Kla and H3K18 lactylation was investigated by Western blotting. **I** Analysis of Pan Kla and H3K18 lactylation in HT29 and SW480 cells following CRC/PLT-Exos co-culturing and LINC00183 overexpression. **J** Examination of H3K18 lactylation and Pan Kla in CCC-HIE-2 and CRC cell lines. **K** CUT&Tag tests on HT29 cells using H3K18la antibodies. H3K18la enrichment was found in many genes’ promoter regions (replicate 1). **L** KEGG database analysis for H3K18la-related genes. **M** Flow diagram showing H3K18la’s downstream target genes. **N** In the GDF15, HDAC10, and CXCL1 promoter regions, H3K18la peaks were found in both Cut&Tag datasets. **O** GDF15 mRNA and protein levels in CRC cells after co-incubation with PLT-Exos or overexpression of LINC00183. **P** GDF15 expression in HT29 and SW480 cells treated with varying amounts of 2-DG and oxamate was examined using Western blot technology. **Q** IF study of GDF15 expression in tissue microarrays comparing CRC samples to matched normal colon tissue. 200 μm scale bar. **R** Prognostic analysis of GDF15 in 93 CRC cases from our center. **S** and **T** GDF15-deficient CRC cells were tested for viability using the CCK-8 assay. **U** Results of colony formation assays conducted in GDF15-deficient CRC cells. **V** The Transwell migration assay was used to assess the GDF15-deficient CRC cells’ capacity for migration. **W**, **X** Correlation between H3K18la and GDF15 expression. Data are derived from IF analysis of CRC tissue microarrays.

To further investigate whether LINC00183 promotes glycolytic activity through ENO1 in CRC cells, we constructed an ENO1-targeting shRNA (sh-ENO1) and an ENO1 overexpression plasmid. ECAR analysis in HT29 and SW480 cells showed that ENO1 overexpression reversed the inhibitory effect of LINC00183 silencing on glycolytic function. Conversely, sh-ENO1 transfection significantly attenuated the increase in glycolytic flux induced by LINC00183 overexpression (Fig. 7A, B and Supplementary Fig. S3B–E). Confirming the positive impact of the LINC00183-ENO1 interaction on the glycolytic capacity of CRC cells, ENO1 overexpression reversed the suppression of lactate production, ATP generation, and glucose consumption mediated by LINC00183 silencing, whereas sh-ENO1 transfection significantly reversed the pro-glycolytic effects induced by LINC00183 overexpression (Fig. 7C and Supplementary Fig. S3D–G, and H–O).

Lactate-induced histone lysine lactylation constitutes an epigfenetic modification that stimulates gene transcription by promoting chromatin accessibility [21]. To investigate the relationship between glycolysis and pan lysine lactylation (PanKIa) and histone lactylation levels in CRC cells, HT29 and SW480 cells were treated with 2-deoxy-D-glucose (2-DG), a glycolysis inhibitor, and oxamate, a lactate dehydrogenase (LDH) inhibitor. Western blot assays showed that 2-DG and oxamate decreased total lysine lactylation (Pan Kla) and H3K18 lactylation in a dose-dependent manner (Fig. 7E–G), while a similar effect was observed upon ENO1 knockdown (Fig. 7H). We postulated that exosomal LINC00183 may be involved in histone lactylation in colorectal cancer (CRC) given its impact on glycolysis and lactate generation.

As shown in Fig. 7I, co-incubation with CRC/PLT-Exos and overexpression of LINC00183 independently and significantly increased global and H3K18 lactylation levels. Notably, CRC cell lines showed abnormally high levels of global lactylation and H3K18 lactylation, which may have a function in carcinogenesis.(Fig. 7J).

To investigate the potential role of histone lactylation in the development of colorectal cancer, we reduced endogenous LDHA and LDHB (Supplementary Fig. S4A) to lower global lactylation and H3K18 lactylation levels. We next carried out rescue studies by adding sodium lactate to CRC cells. Histone lactylation significantly boosted CRC cell proliferation and colony formation ability, according to CCK-8 and colony formation assays (Supplementary Fig. S4B, C, and D). Furthermore, HT29 and SW480 cells’ capacity to migrate was likewise hampered by the concurrent reduction of LDHA and LDHB (Supplementary Fig. S4E). Crucially, sodium lactate supplementation lessened the inhibition of CRC cell migration (Supplementary Fig. S4E) and proliferation (Supplementary Fig. S4B, C, and D) brought on by LDHA/B deficit. These findings imply that H3K18 lactylation is promoted by LINC00183 in CRC.

### GDF15 functions as an oncogene in colorectal cancer and is a target of H3K18 lactylation

Using ChIP-grade H3K18la antibodies, we conducted two separate CUT&Tag assays in HT29 cells to clarify the regulation mechanism of H3K18 lactylation(Supplementary Files 5 and 6). As shown in Fig. 7K and Supplementary Fig. S5A, several genes had H3K18la abundant in their promoter regions. Furthermore, our CUT&Tag sequencing of genes linked to H3K18la was enriched in multiple signaling pathways implicated in CRC carcinogenesis, according to KEGG database analysis(Fig. 7L and Supplementary Fig. S5B).

Next, we integrated transcriptome data comparing gene expression profiles in control and LINC00183-overexpressing HT29 cells with CUT&Tag data, differential gene expression data from the TCGA COAD cohort, and the PubMed database and identified GDF15, HDAC10, and CXCL1 as candidate H3K18la-regulated genes (Fig. 7M and Supplementary File 7). Analysis of our CUT&Tag data indicated H3K18la enrichment in the promoter regions of all three genes (Fig. 7N). Subsequently, we validated the mRNA expression levels of these target genes in CRC cells, with results showing that GDF15 was significantly downregulated by LINC00183 knockdown(Supplementary Fig. S5C and D). On subsequent analysis, we confirmed that GDF15 mRNA and protein levels were upregulated in HT29 and SW480 cells following independent CRC/PLT-Exos co-incubation and overexpression of LINC00183 **(**Fig. 7O). Consistent with the above findings, the addition of glycolysis inhibitors negatively regulated GDF15 protein expression in CRC cells (Fig. 7P).

GDF15 has been recently identified as a promoter of tumor progression [22**–**26]. Following CRC microarray research, we discovered that, in contrast to normal colon tissues, GDF15 expression was increased in CRC tissues (Fig. 7Q), and was closely associated with poor tumor prognosis (Fig. 7R). Lastly, we looked at the biological roles of GDF15 in HT29 and SW480 cells that were GDF15-deficient. Depletion of GDF15 significantly reduced tumor cell migration, colony formation, and proliferation, as seen in Fig. 7S-V. Additionally, there was a strong positive association found between the levels of GDF15 protein expression and H3K18la (Fig. 7W and X). In conclusion, our research shows that GDF15 functions as an oncogene in human colorectal cancer and is directly controlled by H3K18 lactylation.

### Lactylation of H3K18 stimulates the growth of CRC via expressing GDF15

We next performed rescue experiments to verify the relationship between H3K18 lactylation and GDF15 in CRC progression. In particular, we knocked down LDHA and LDHB to lower histone lactylation levels, and then we restored GDF15 in LDH-deficient colorectal cancer cells. Results showed that GDF15 partially counteracted the suppressive effects of LDHA/B silencing on the viability (Supplementary Fig. S6A) and the colony formation (Supplementary Fig. S6B), and the migration (Supplementary Fig. S6C) capacities of CRC cells.

### LINC00183-dependent ENO1 stabilization promotes growth and metastasis of CRC

**Fig. 8:**
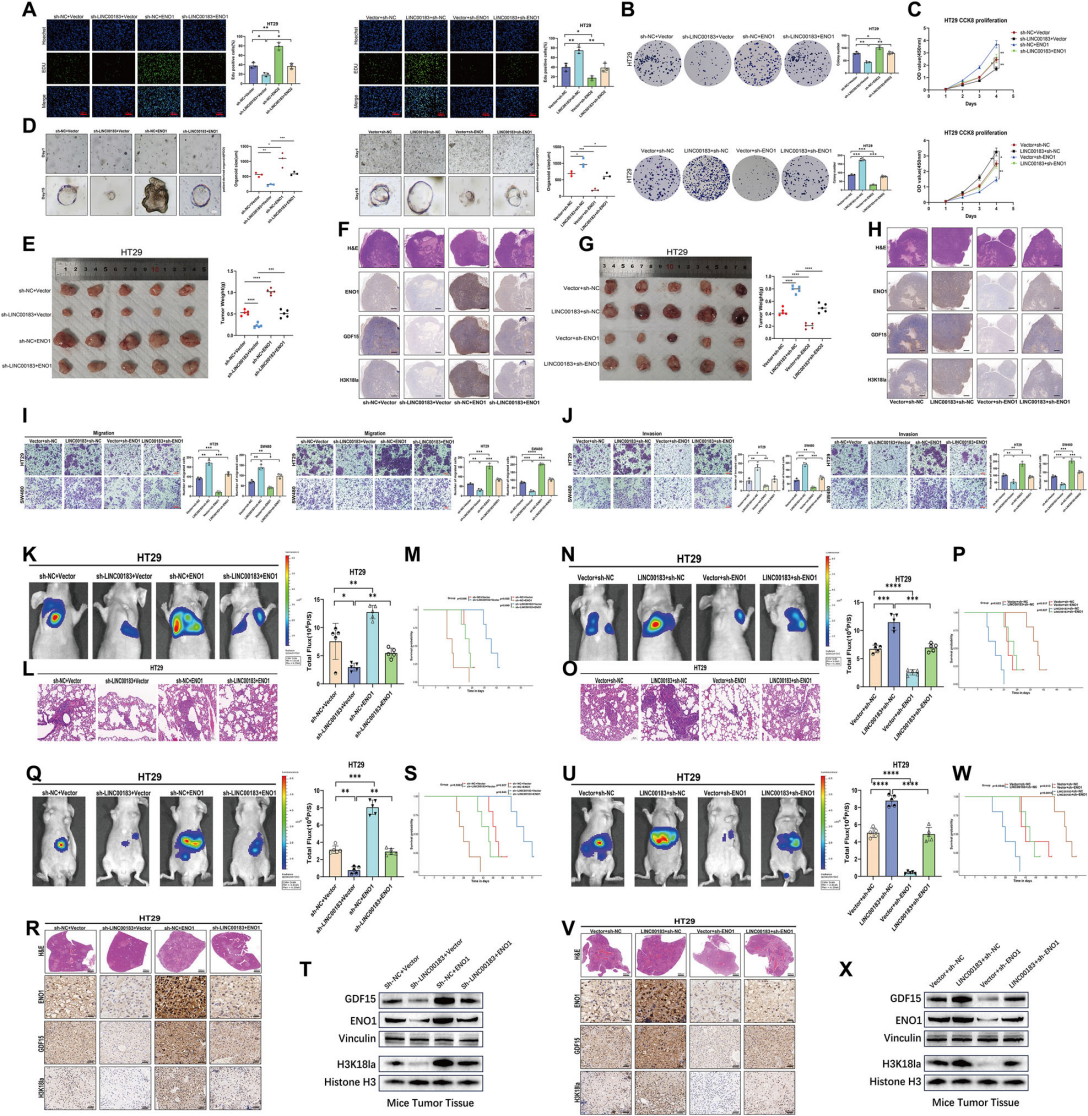
LINC00183-mediated ENO1 stabilization promotes CRC growth and metastasis. Human CRC cells were transfected with sh-LINC00183, sh-ENO1, LINC00183 overexpression plasmids, or ENO1 overexpression plasmids. Results of EdU assays (**A**), colony formation assays (**B**), and CCK-8 assays (**C**). **D** Effect of ENO1/LINC00183 knockdown/overexpression in CRC PDOs. **E** and **G** Representative images of mice bearing subcutaneous HT29 cell xenografts, and corresponding tumor weight measurements. Data represent mean ± SEM from five independent samples. **F**, **H** Representative H&E staining and ENO1, GDF15, and H3K18la IHC images from tumor-bearing mice. Scale bars, 200 μm. **I** and **J** Results of Transwell assays performed in HT29 and SW480 cells. **K**–**P** Representative in vivo bioluminescence and lung H&E staining (n = 5) images, survival curves, and corresponding statistical analyses. **Q–X** Representative in vivo bioluminescence and liver H&E staining and IHC images (*n* = 5), survival curves, and protein levels in liver metastases as measured by immunoblotting.

We evaluated the effects of ENO1 overexpression and knockdown in CRC cells, both in vitro and in vivo to further validate that LINC00183 stimulates CRC development and metastasis via ENO1. Results obtained from EdU cell proliferation assays, colony formation assays, CCK8 assays, PDO models, and the CRC subcutaneous xenograft mouse model showed that ENO1 overexpression reversed the inhibition of CRC cell growth induced by LINC00183 silencing, while ENO1 knockdown significantly attenuated the pro-proliferative phenotype driven by LINC00183 overexpression (Fig. 8A–D, E and G)(Supplementary Fig. S7A–D). Furthermore, we used IHC to measure the expression levels of ENO1, GDF15, and H3K18la in subcutaneous colorectal cancer tumors from various treatment groups; the results were in line with earlier studies(Fig. 8F–H). These findings demonstrate that LINC00183 promotes the proliferation of human CRC cells by enhancing H3K18 lactylation through the key glycolytic enzyme ENO1.

We also carried out a number of in vitro and in vivo studies to look at how the LINC00183-ENO1 axis contributes to tumor metastasis. Results showed that in Transwell migration and invasion assays, wound-healing assays, as well as in the mouse lung metastasis and spleen injection liver metastasis models employed in this study, ENO1 overexpression reversed the inhibitory effect of sh-LINC00183 on the metastatic potential of CRC cells, while sh-ENO1 transfection significantly attenuated the pro-metastatic phenotype driven by LINC00183 overexpression (Fig. 8I–L, N, O, Q, and U)(Supplementary Fig. S7E, F). The corresponding survival curves (Fig. 8M, P, S, and W) were consistent with these findings. Additionally, we used IHC and western blotting to evaluate the expression levels of ENO1, GDF15, and H3K18la in the liver metastases, and the results were in line with earlier studies(Fig. 8R, T, V, and X). These findings further suggest that metastatic dissemination of human CRC cells is facilitated by enhanced H3K18 lactylation secondary to LINC00183-mediated ENO1 stabilization.

### Effectiveness of antiplatelet drugs in combination with chemotherapy in animal models of CRC

The experimental results described so far suggest that platelet-derived exosomal LINC00183 promotes growth and metastasis in CRC. Studies have identified the P2Y12 pathway as the primary route of ADP-mediated platelet activation. Activated platelets release large quantities of exosomal vesicles, soluble factors, and associated lncRNAs [27], which may protect cancer cells from chemotherapy-induced apoptosis and contribute to poor chemotherapy outcomes [28], thus, identifying effective antiplatelet drugs holds significant translational potential.

**Fig. 9:**
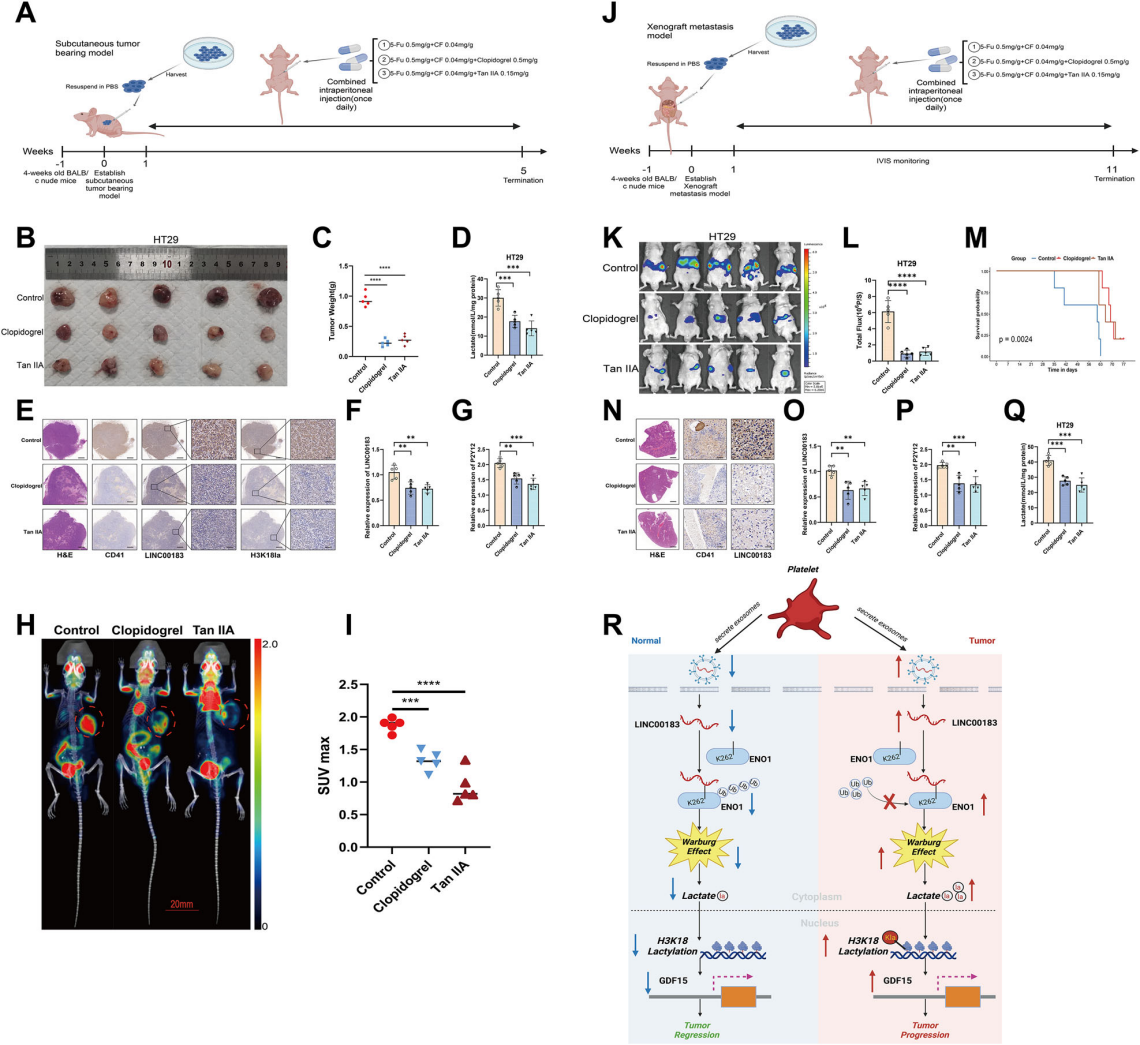
Evaluation of the effectiveness of antiplatelet drugs in combination with chemotherapy in animal models of CRC. **A** The xenograft nude mouse model’s schematic. **B**, **C** A quantitative analysis was conducted on the subcutaneous xenograft model and tumor weight. **D** Measurement of lactate levels in subcutaneous tumor tissue. **E** Representative H&E staining, FISH analysis of LINC00183 expression, and CD41 and H3K18la IHC analysis in excised CRC xenografts. **F**, **G** RT-qPCR-based quantification of LINC00183 and P2Y12 mRNA in sera from mice bearing CRC xenografts. **H** and **I** PET-CT-based assessment of glucose uptake in subcutaneous tumors. **J** Schematic representation of the chemotherapy/antiplatelet regimens used in the splenic injection model for liver metastasis. **K** Representative images of bioluminescent in vivo detection of liver metastases. **L** Total photon flux quantification data. **M** OS curves. **N** Representative H&E-staining images, FISH analysis of LINC00183 expression, and CD41 IHC analysis in excised livers. **O**, **P** RT-qPCR-based quantification of LINC00183 and P2Y12 mRNA in sera from mice with CRC liver metastases. **Q** Quantification of lactate levels in excised liver tissue. **R** Schematic representation of main findings of the current study. LINC00183-induced CRC progression is driven by stabilization of the key glycolytic enzyme ENO1, with consequent H3K18 lactylation and enhanced GDF15 transcription.

We selected two P2Y12 receptor antagonists, clopidogrel [29] and tanshinone IIA (Tan IIA) [30], and investigated their efficacy, in combination with the FOLFIRI chemotherapy regimen, in our CRC xenograft and CRC liver metastasis mouse models. Chemotherapy administration was strictly calibrated based on mouse body surface area. For the standard clinical FOLFIRI regimen, 5-Fu (0.5 mg/g) and CF (0.04 mg/g) were administered by intraperitoneal injection. Based on standard clinical dosage of clopidogrel (75–300 mg/g) and Tan IIA (40–80 mg/g), the corresponding doses for mice varied between 0.19–0.75 mg/g and 0.1–0.2 mg/g, respectively (Fig. 9A and J). In mice bearing CRC xenografts, compared with the chemotherapy-only (FOLFIRI) group the combination of clopidogrel or Tan IIA with chemotherapy significantly reduced tumors’ weight and lactate contents (Fig. 9B–D). IHC and FISH analysis of tumor sections revealed a marked reduction in CD41-positive platelet aggregation, LINC00183 expression, and H3K18 lactylation in the combination chemotherapy groups (Fig. 9E). Serum levels of P2Y12 and LINC00183 also showed statistically significant intergroup differences (Fig. 9F, G). Furthermore, PET-CT imaging showed that the tumors in the combination treatment groups had much less glycolytic activity (Fig. 9H, I).

In the spleen injection liver metastasis model, the combination of clopidogrel or Tan IIA with chemotherapy significantly reduced the number of metastatic nodules and improved survival prognosis, in comparison to the chemotherapy-only group (Fig. 9K–M). Similar to the xenograft model, IHC and FISH analysis of liver sections revealed a significant reduction of CD41-positive platelet aggregation and LINC00183 expression in the combination chemotherapy groups (Fig. 9N). Also in line with findings in the xenograft model, serum levels of P2Y12 and LINC00183, as well as lactate levels in liver tumors, also exhibited statistically significant intergroup differences (Fig. 9O–Q). Additionally, we have confirmed that the expression levels of LINC00183, ENO1, H3K18la, and GDF15 in tissue microarrays (TMAs) correlate with those of CD41-positive platelets. Immunofluorescence staining was performed to visualize representative co-staining images of CD41, H3K18la, and GDF15 in samples with high and low CD41 expression(Supplementary Fig. S8A). According to the TMA, the CD41-high expression group had significantly greater expression levels of LINC00183, ENO1, H3K18la, and GDF15 in CRC tumor tissues than the CD41-low expression group (Supplementary Fig. S8B-E).

These findings suggest that inhibiting platelet activation and exosome release with drugs such as clopidogrel or Tan IIA may, in combination with chemotherapy, significantly reduce the source of LINC00183 and improve patient prognosis.

## Discussion

In this study, through comparative high-throughput RNA-seq of PLT-Exos from CRC patients and healthy controls, we identified lncRNA, LINC00183, associated with CRC progression. Our findings demonstrate that LINC00183 acts as an oncogene in CRC. It is correlated with malignant phenotype, stage advancement, and lower survival rates, and its expression is noticeably higher in CRC tissue than in normal colon tissue. Using in vitro and in vivo assays, we provide evidence that exosomal LINC00183 is transferred to CRC cells, where it binds to and stabilizes the glycolytic enzyme ENO1 by inhibiting its degradation through the ubiquitin-proteasome pathway. This leads to enhanced aerobic glycolysis, resulting in lactate accumulation, H3K18 lactylation, and oncogenic GDF15 expression. These findings imply that exosomal LINC00183 may be a viable therapeutic target and uncover a novel functional role for this lncRNA in the development of colorectal cancer.

Exosomes are sub-micron, spherical membrane-bound structures secreted by prokaryotic and eukaryotic cells [31**–**33]. Their lipid bilayer membrane protects their cargo from enzymatic degradation by proteases and ribonucleases [34]. Platelets have the expression of two main G protein-coupled receptors, P2Y1 and P2Y12. The P2Y12 pathway is the primary route of ADP-mediated platelet activation, which results in the release of large quantities of exosomal vesicles, soluble factors, and associated lncRNAs [27]. According to research, platelets are essential for preventing chemotherapy-induced apoptosis in cancer cells and preserving the integrity of the tumor vasculature, both of which lead to poor chemotherapy outcomes [28]. Exosomes are abundant in peripheral blood in both healthy persons and those with a variety of diseases. Red blood cells, white blood cells, platelets, and endothelial cells all release exosomes into the bloodstream [35]. In the plasma of healthy individuals, most of these exosomes (70–90%) are derived from activated or apoptotic platelets [36**–**38]. The abundance of circulating PLT-Exos is significantly increased in patients with different cancer types, including glioblastoma, gastric cancer, lung cancer, and melanoma, suggesting their potential as diagnostic biomarkers [39]. Evidence indicates that PLT-Exos can easily traverse the vascular endothelium, infiltrate and alter the TME, and interact with tumor cells to facilitate their proliferation and metastasis [40]. In many instances, these pro-tumor functions are associated with the transfer of specific cargo molecules from exosomes to recipient cells. Bakewell et al. showed that platelet glycoprotein IIb/IIIa antagonists reduce the formation of bone metastases by B16 melanoma cells by inhibiting the interaction between cancer cells and platelets [41]. In the lung cancer cell line A549, exosomal CD41 enhances exosome adhesion to fibrinogen and human umbilical vein endothelial cells (HUVECs), resulting in significant stimulation of cancer cell migration [42]. Furthermore, it has been demonstrated that exosome contact with lung cancer cells activates the AKT signaling pathway and mitogen-activated protein kinase (MAPK) p42/44, all of which are involved in responses to cell growth [42]. Ingrid Fleming et al. confirmed that PLT-Exos contain miR-126 and miRNA-223, which are key players in tumorigenesis. miRNA-126 stimulates VEGF-dependent endothelial cell proliferation, while miRNA-223 inhibits angiogenesis by targeting endothelial β1 integrin [43]. A study by Tang et al. revealed that the transfer of platelet-derived exosomal miR-939 promoted invasive behavior in cultured ovarian epithelial carcinoma cells by inducing epithelial-mesenchymal transition (EMT) [44]. Meanwhile, Yao et al. reported that tropomyosin 3 (TPM3) mRNA is overexpressed in platelets from breast cancer patients and is transferred to breast cancer cells via exosomes to promote tumor invasion [45].

Metabolic reprogramming is a hallmark of tumorigenesis, with cancer cells often and preferentially engaging in anaerobic glycolysis even under aerobic conditions (Warburg effect) to rapidly generate energy and metabolic intermediates [46]. However, the mechanisms by which tumor-associated and circulating platelets regulate tumor glycolysis and progression remain incompletely understood. Exosomes are rich in non-coding RNAs such as mi-RNAs and lncRNAs, derived from their cells of origin [47]. Evidence shows that diverse non-coding RNA species, including small nuclear RNAs and miRNAs, encapsulated in exosomes can be efficiently transported to target cells to regulate their functions [48, 49]. Our study demonstrated that platelets from CRC patients release exosomes containing the lncRNA LINC00183, which promotes in CRC cells aerobic glycolysis, proliferation, and invasiveness by preventing ENO1 degradation. By accelerating the glycolysis pathway’s transformation of 2-phosphoglycerate into phosphoenolpyruvate, ENO1 plays a critical function in cellular energy metabolism. ENO1 is a multifunctional protein with oncogenic properties, as it was shown to drive the progression of various cancer types by promoting tumor cell proliferation, migration, and invasion [17]. Among the four isoforms of enolase, ENO1 is widely expressed in most human tissues and is overexpressed in various cancer types [50].

PTMs of histones play a crucial role in regulating gene expression by serving as binding sites to recruit chromatin regulatory factors, thereby modulating a variety of biological processes [51, 52]. Typically, the N-terminal tails of histones undergo numerous PTMs, such as methylation, acetylation, and succinylation [53]. In 2019, Zhang et al. discovered that histone lactylation is an epigenetic alteration that directly stimulates gene transcription from chromatin and is reliant on intracellular lactate generated by glycolysis or other metabolic processes [21]. Unlike normal cells, tumor cells preferentially undergo glycolysis over oxidative phosphorylation under aerobic conditions to generate sufficient energy for their rapid growth and survival. This phenomenon, known as the Warburg effect, is a fundamental metabolic characteristic of cancer cells [46]. The Warburg effect leads to lactate accumulation within tumor cells, resulting in abnormal levels of histone lactylation. Dysregulation of various histone modifications is commonly observed in a wide range of human diseases, including cancer [54]. Lin et al. revealed that histone lactylation drives the initiation and progression of CRC by promoting CRC cell survival under hypoxic conditions, enhancing in turn, resistance to bevacizumab therapy [55]. According to Fan et al., histone lactylation accelerates the development of uveal melanoma by upregulating YTHDF2, which identifies and enhances the degradation of m6A-modified PER1 and TP53 mRNAs [56]. According to Li et al., H3K18 lactylation promotes cisplatin resistance in bladder cancer by driving the expression of the transcription factors YBX1 and YY1, and inhibiting H3K18 lactylation can restore cisplatin sensitivity [57]. These investigations demonstrate the important epigenetic regulatory function of histone lactylation in the development of tumors.

Moreover, recent studies have highlighted the pivotal role of the gut microbiome in modulating cancer initiation, progression, and therapeutic response [58, 59]. Specific microbial species and their metabolites can influence the expression of non-coding RNAs (such as miRNAs and lncRNAs), thereby participating in the regulation of oncogenic and tumor-suppressive pathways. In colorectal cancer, alterations in the composition of the gut microbiota may affect the production of exosomes and the stability and function of their RNA cargo, indirectly modulating the oncogenic effects of key lncRNAs such as LINC00183. Therefore, future investigations should explore how the gut microbiota mediates the role of exosomal lncRNAs in tumor progression, which may offer novel insights and therapeutic avenues for targeting LINC00183 in clinical settings.

In conclusion, we report for the first time that the lncRNA LINC00183 is differentially expressed in PLT-Exos from CRC patients and can be delivered to CRC cells to promote tumor growth and metastasis. Our findings also provide compelling evidence that the pro-tumoral effect of LINC00183 is linked to enhanced glycolysis via binding and stabilization of ENO1, leading to H3K18 lactylation and transcriptional activation of the oncogene GDF15. These findings highlight a novel mechanism contributing to CRC progression and suggest the potential of LINC00183 as a new prognostic marker and therapeutic target. Moreover, since LINC00183 can be carried by platelet-derived exosomes and released into the circulation, it offers a convenient, non-invasive detection method. Its expression level holds promise as an early diagnostic biomarker in liquid biopsy. Furthermore, therapeutic strategies targeting LINC00183 or its downstream regulatory axis may provide novel personalized treatment options for colorectal cancer patients, particularly those with resistance or relapse following standard therapies.

### Reporting summary

Further information on research design is available in the Nature Research Reporting Summary linked to this article.

## Supplementary information

Supplementary Figure legends

Supplementary Figure S1

Supplementary Figure S2

Supplementary Figure S3

Supplementary Figure S4

Supplementary Figure S5

Supplementary Figure S6

Supplementary Figure S7

Supplementary Figure S8

WB-raw data

Supplementary File1

Supplementary File2

Supplementary File3

Supplementary File4

Supplementary File5

Supplementary File6

Supplementary File7

Supplementary Table

## Data Availability

All data generated or analyzed during this study are included in this published article and its supplementary files. The TCGA dataset is available from the UCSC Xena platform (https://xenabrowser.net/). The data reported in this study have been deposited in the OMIX database (https://ngdc.cncb.ac.cn/omix; accession number: OMIX010195) and the GSA-Human database (https://ngdc.cncb.ac.cn/gsa-human; accession number: HRA011551, HRA011676) at the China National Center for Bioinformation / Beijing Institute of Genomics, Chinese Academy of Sciences. Processed data are available from the corresponding author upon reasonable request.
