# Supplementary material for: Platelet-derived exosomal LINC00183 facilitate colorectal cancer malignant progression driven by histone lactylation through stabilizing ENO1
Source: Cell Death Dis. 2025 Aug 7;16(1):593. doi: 10.1038/s41419-025-07914-4 (PMC12331901; doi:10.1038/s41419-025-07914-4)
Supplement: Supplementary file 1 — Supplementary Figure legends [file 41419_2025_7914_MOESM1_ESM.docx]

**Supplementary Figure**

**Supplementary Figure S1**

**Supplementary Figure S1.** Platelet-derived exosomes promote the tumorigenesis of CRC cells in vitro. Cultured CRC cells were incubated with platelet-derived exosomes purified from plasma of CRC patients. **A** and **B,** CRC cell viability was measured by CCK-8 assays. **C** and **D,** Results of EdU proliferation assays. Scale bar,  50 μm. **E** and **F,** Transwell migration and invasion assays conducted in HT29 and SW480 cells. Scale bar,  50 μm. **G** and **H,** Results of wound-healing assays conducted in HT29 and SW480 cells. Scale bar, 50 μm. **I** and **J,** Tumor cells were co-cultured with platelets in a microchamber. LINC00183 level was measured by RT-PCR.

**Supplementary Figure S2**

**Supplementary Figure S2. A** and **B,** RT-qPCR analysis of relative LINC00183 expression in HT29 and SW480 cells transfected with the indicated si-RNAs or plasmids to verify transfection efficiency. **C,** RNA-FISH analysis revealed that LINC00183 is distributed in the cytoplasm and nucleus of HT29 and SW480 cells. Scale bar, 20 μm. **D** and **E,** Determination of ENO1 ubiquitination in SW480 cells co-transfected with His-tagged ubiquitin, FLAG-tagged ENO1, and either si-LINC00183, LINC00183 plasmid, or corresponding controls. Subsequently, the SW480 cells were treated with MG132 (10 μmol/L) for 10 hours. Following cell lysis, a Co-IP assay was performed using anti-FLAG antibodies for precipitation, and western blot analysis was conducted with anti-His antibodies. TCL: total cell lysate. **F,** SW480 cells were transfected with plasmids expressing the indicated FLAG-tagged wild-type or mutant ENO1 (K224-227A, K228A, K262A, and K281A) along with His-tagged ubiquitin plasmids, and specific si-RNA targeting LINC00183 or the corresponding control. Following a 10-hour treatment with MG132 (10 μmol/L), Co-IP and western blot analyses were conducted to assess the ubiquitination levels of wild-type and mutant FLAG-ENO1. **G-I,** HT29 and SW480 cells were transfected with plasmids expressing FLAG-tagged ENO1 or FLAG-tagged mutant ENO1 (K224-227A, K228A, and K281A). Subsequently, a lncRNA pull-down assay was performed using the LINC00183 probe, and western blot analysis was conducted using a specific FLAG antibody.

**Supplementary Figure S3**

**Supplementary Figure S3. A,** Schematic representation of glycolysis. The enolase family is marked in red. **B-E,** Extracellular acidification rate (ECAR) was assessed using a Seahorse XF Extracellular Flux Analyzer following ENO1/LINC00183 knockdown/overexpression in HT29 cells. **F** and **G,** Results of lactate production assays. **H-K,** ATP synthesis measurements. **L-O,** Results of glucose consumption assays.

**Supplementary Figure S4**

**Supplementary Figure S4.** H3K18 lactylation promotes CRC progression. **A,** Confirmation of LDHA/B knockdown efficiency after transfection with si-LDHA/B. **B** and **C,** A CCK-8 assay was used to determine the viability of LDH-deficient CRC cells. **D,** Results of colony formation assays conducted in LDH-deficient CRC cells. **E,** The migratory ability of LDH-deficient CRC cells was evaluated by Transwell migration assay. All the data are presented as the mean ± standard deviation (n = 3). *P < 0.05, **P < 0.01, compared with the control group.

**Supplementary Figure S5**

**Supplementary Figure S5. A,** CUT&Tag was performed with H3K18la antibodies in HT29 cells. Results showed that H3K18la was enriched in the promoter region of numerous genes (rep2). **B,** KEGG database analysis for H3K18la-related genes (rep2). **C** and **D,** To detect the mRNA levels of GDF15, HDAC10, and CXCL1 in cells with linc00183 knockdown.

**Supplementary Figure S6**

**Supplementary Figure S6.** GDF15 overexpression attenuates antitumor effects induced by glycolysis inhibition. **A,** Cell viability was assessed in control and GDF15-overexpressing LDH-deficient CRC cells. **B,** Results of colony formation assays performed in control and GDF15-overexpressing LDH-deficient CRC cells. **C,** Results of Transwell migration assay performed in control and GDF15-overexpressing LDH-deficient CRC cells. All data are presented as mean ± standard deviation (n = 3). *P < 0.05, **P < 0.01, compared to the control group.

**Supplementary Figure S7**

**Supplementary Figure S7.** LINC00183-mediated ENO1 stabilization promotes CRC growth and metastasis. Human CRC cells were transfected with sh-LINC00183, sh-ENO1, LINC00183 overexpression plasmids, or ENO1 overexpression plasmids. Results of EdU assays (SW480)(**A** and **B**), CCK-8 assays (SW480)(**C** and **D**), and wound-healing assay(**E-H**).

**Supplementary Figure S8**

**Supplementary Figure S8.** Validation of the correlation between the expression levels of CD41-positive platelets and LINC00183, ENO1, H3K18la, and GDF15. **A,** The expression of CD41, H3K18la, and GDF15 in a TMA cohort including 93 CRC cases was detected using immunofluorescence. Representative co-staining images of CD41, H3K18la, and GDF15 detection in high and low CD41 expression samples. Scale bars: 500 µm. **B-E,** The scatter plot illustrates the expression levels of LINC00183, ENO1, H3K18la and GDF15 in CRC tumor tissues from the CD41 high-expression and low-expression groups defined in the TMA.
