# Supplementary figures and images for: Platelet-derived exosomal LINC00183 facilitate colorectal cancer malignant progression driven by histone lactylation through stabilizing ENO1
Source: Cell Death Dis. 2025 Aug 7;16(1):593. doi: 10.1038/s41419-025-07914-4 (PMC12331901; doi:10.1038/s41419-025-07914-4)

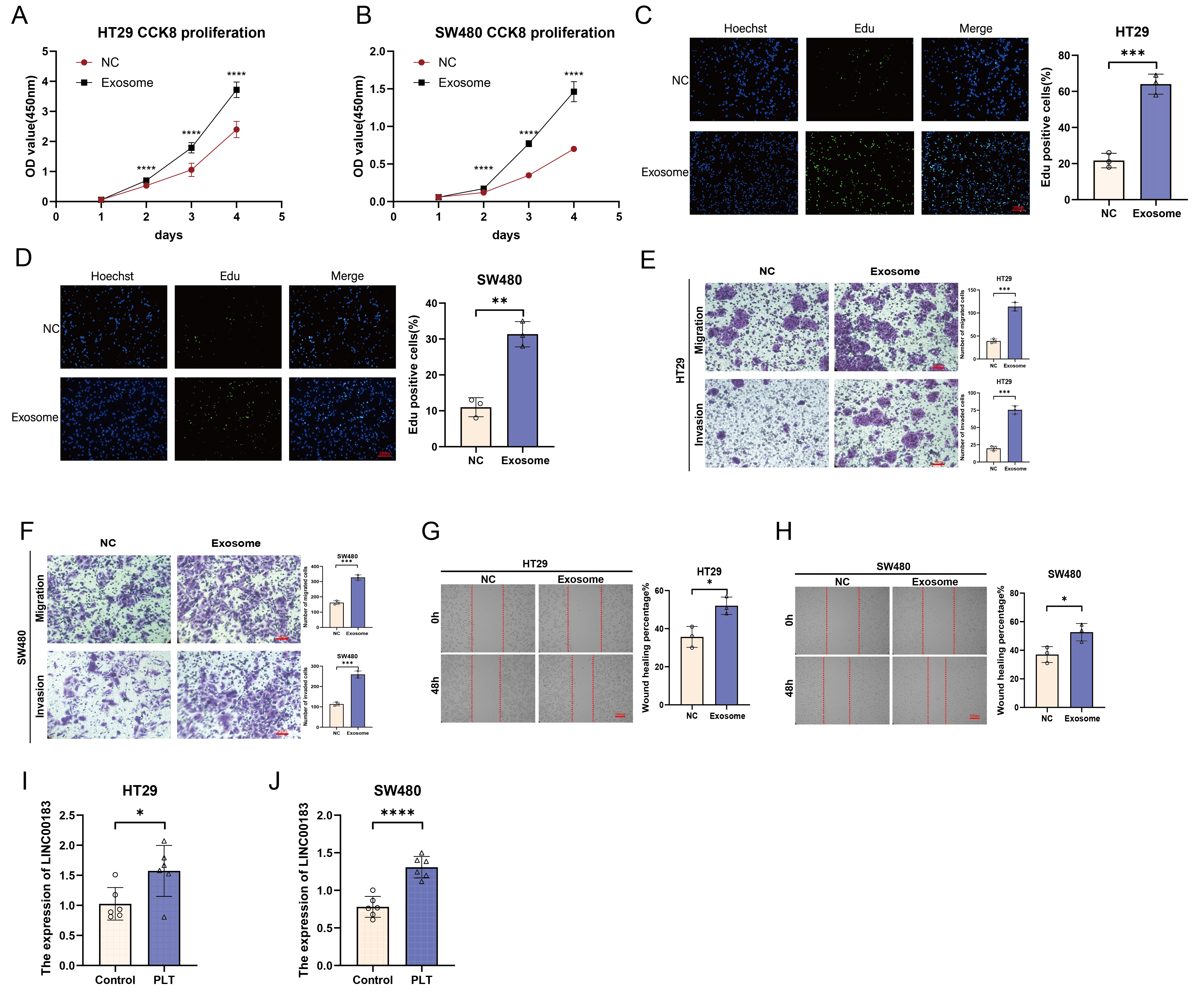

Supplement: Supplementary file 2 — Supplementary Figure S1 [file 41419_2025_7914_MOESM2_ESM.png]

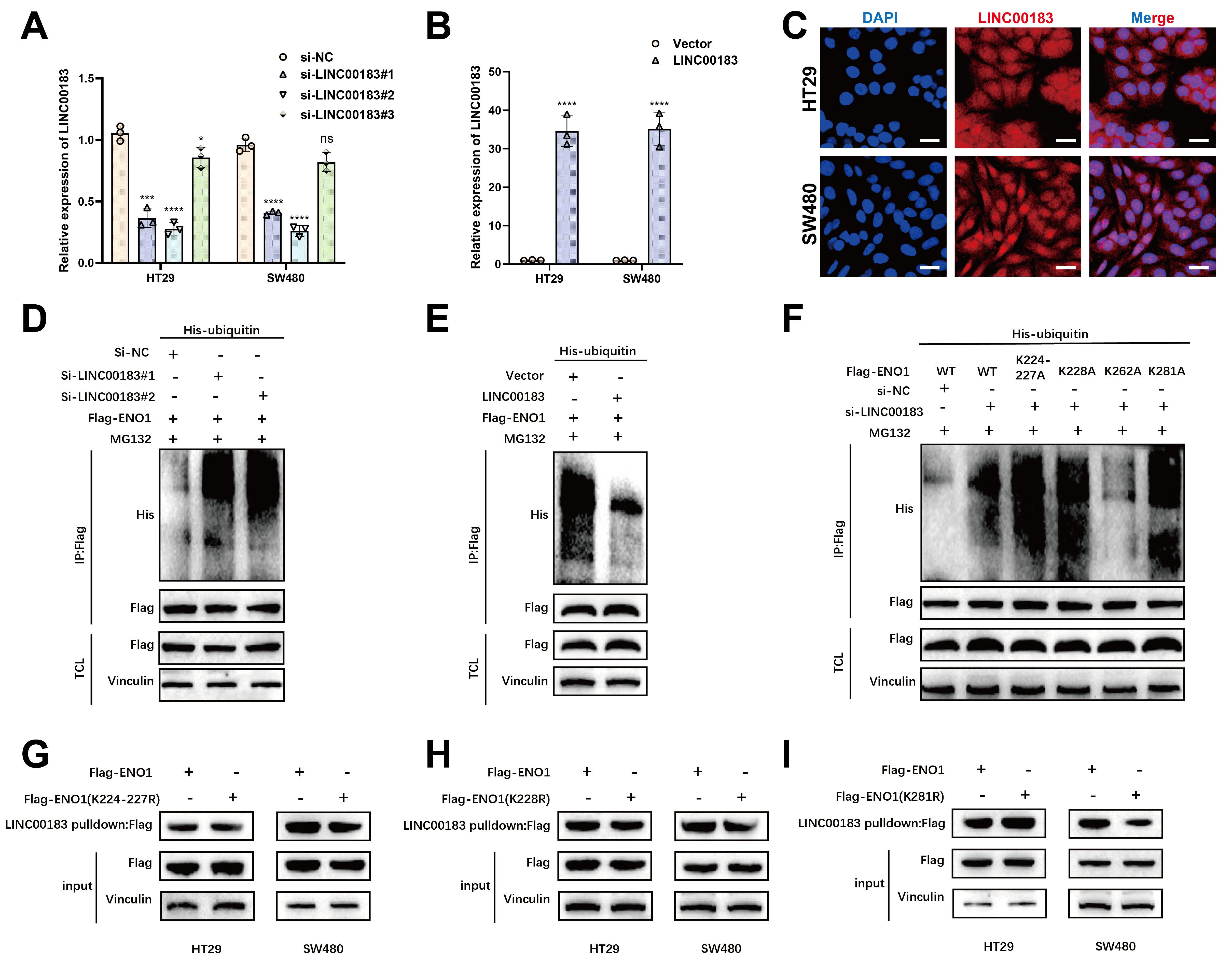

Supplement: Supplementary file 3 — Supplementary Figure S2 [file 41419_2025_7914_MOESM3_ESM.png]

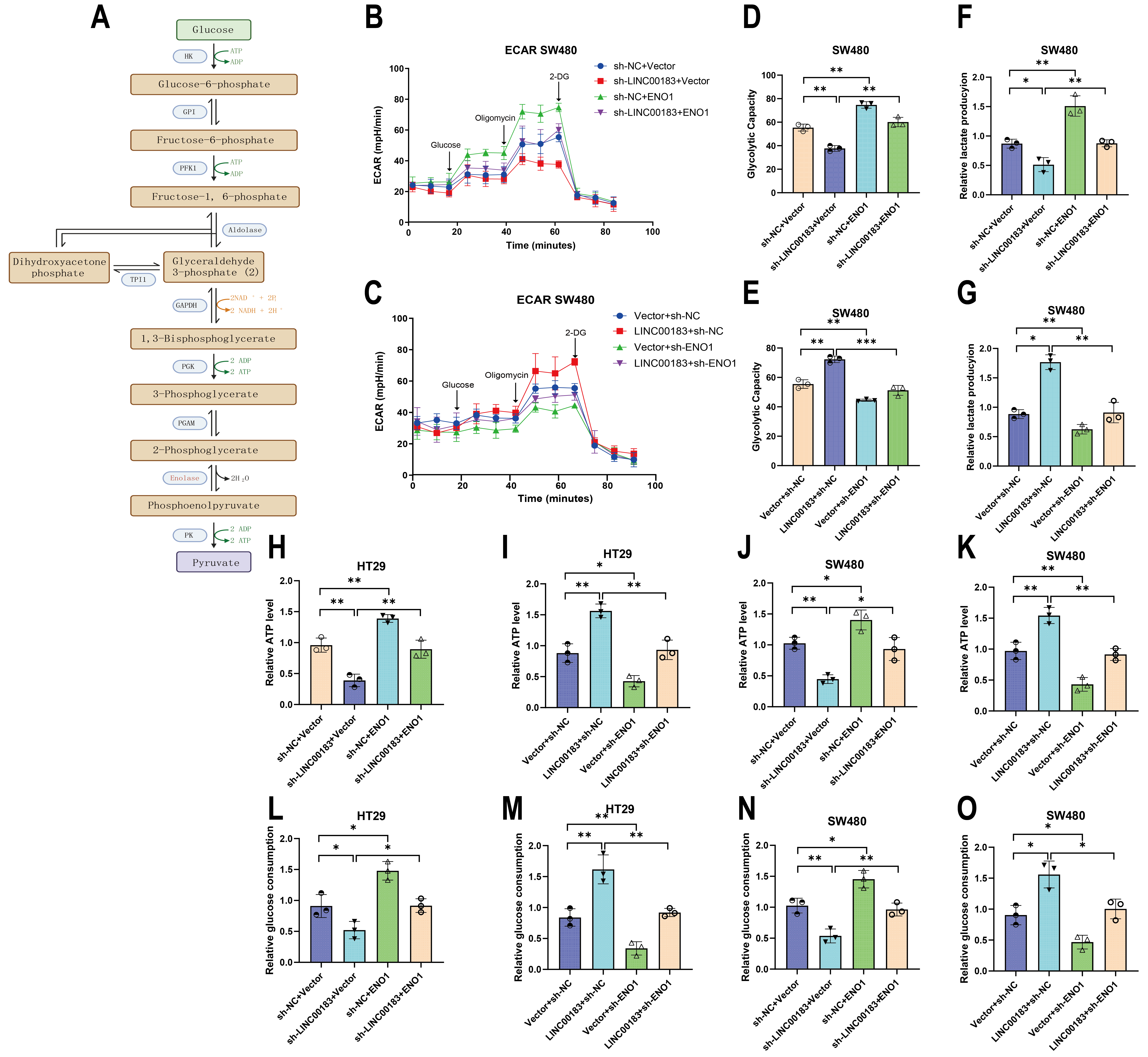

Supplement: Supplementary file 4 — Supplementary Figure S3 [file 41419_2025_7914_MOESM4_ESM.png]

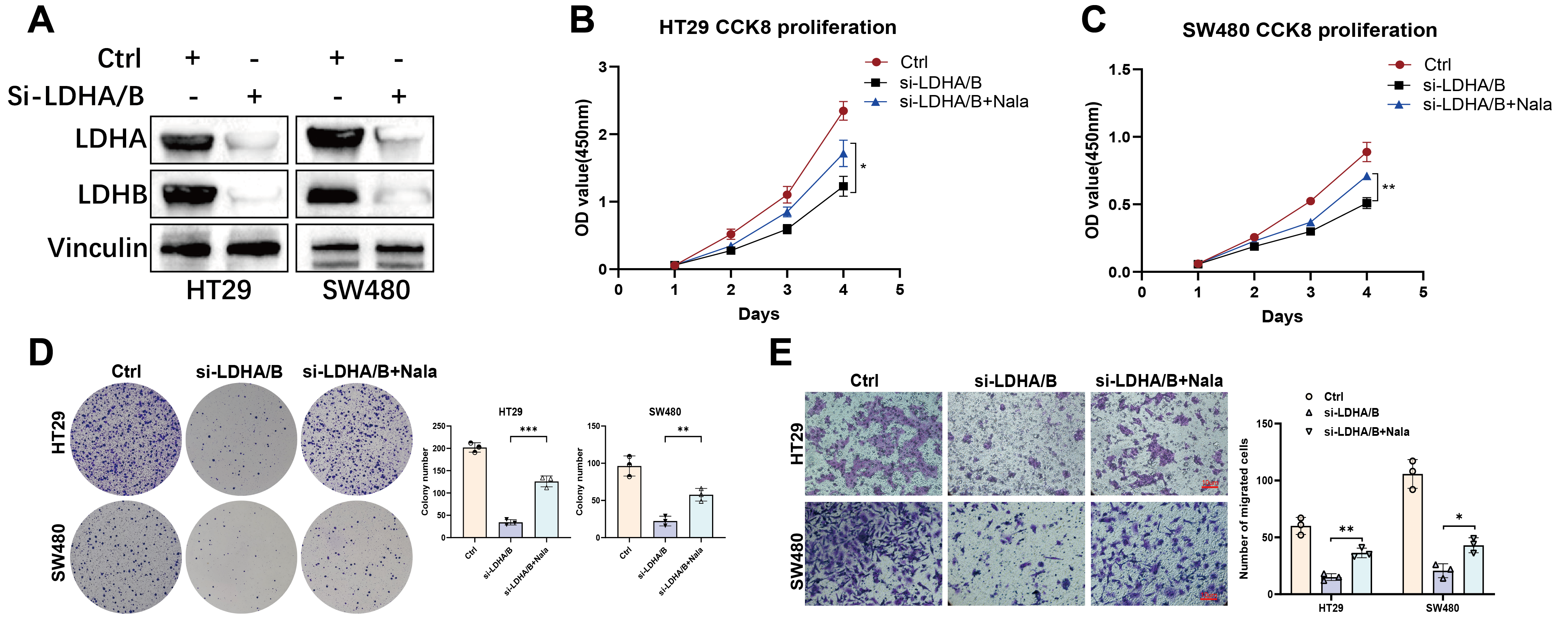

Supplement: Supplementary file 5 — Supplementary Figure S4 [file 41419_2025_7914_MOESM5_ESM.png]

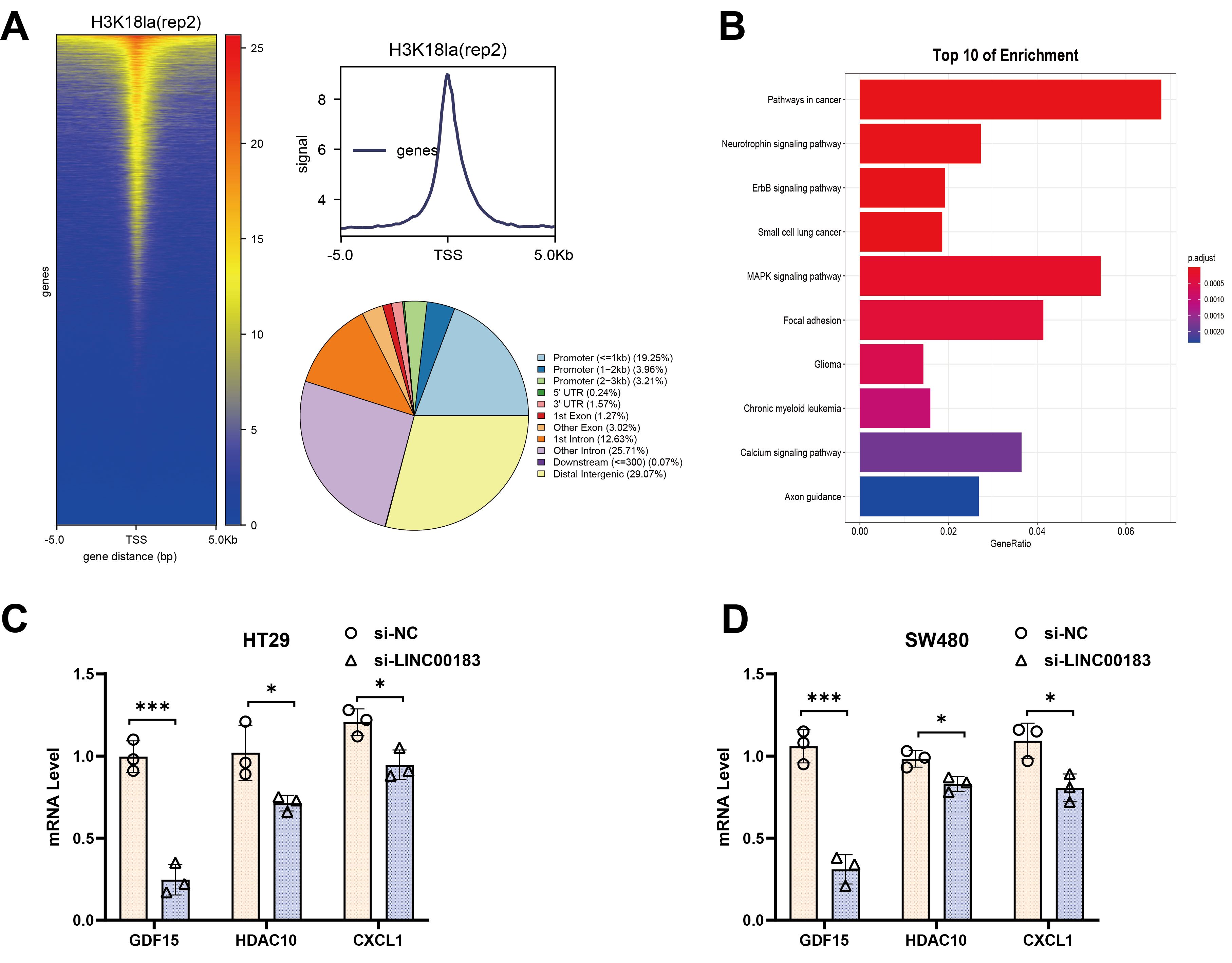

Supplement: Supplementary file 6 — Supplementary Figure S5 [file 41419_2025_7914_MOESM6_ESM.png]

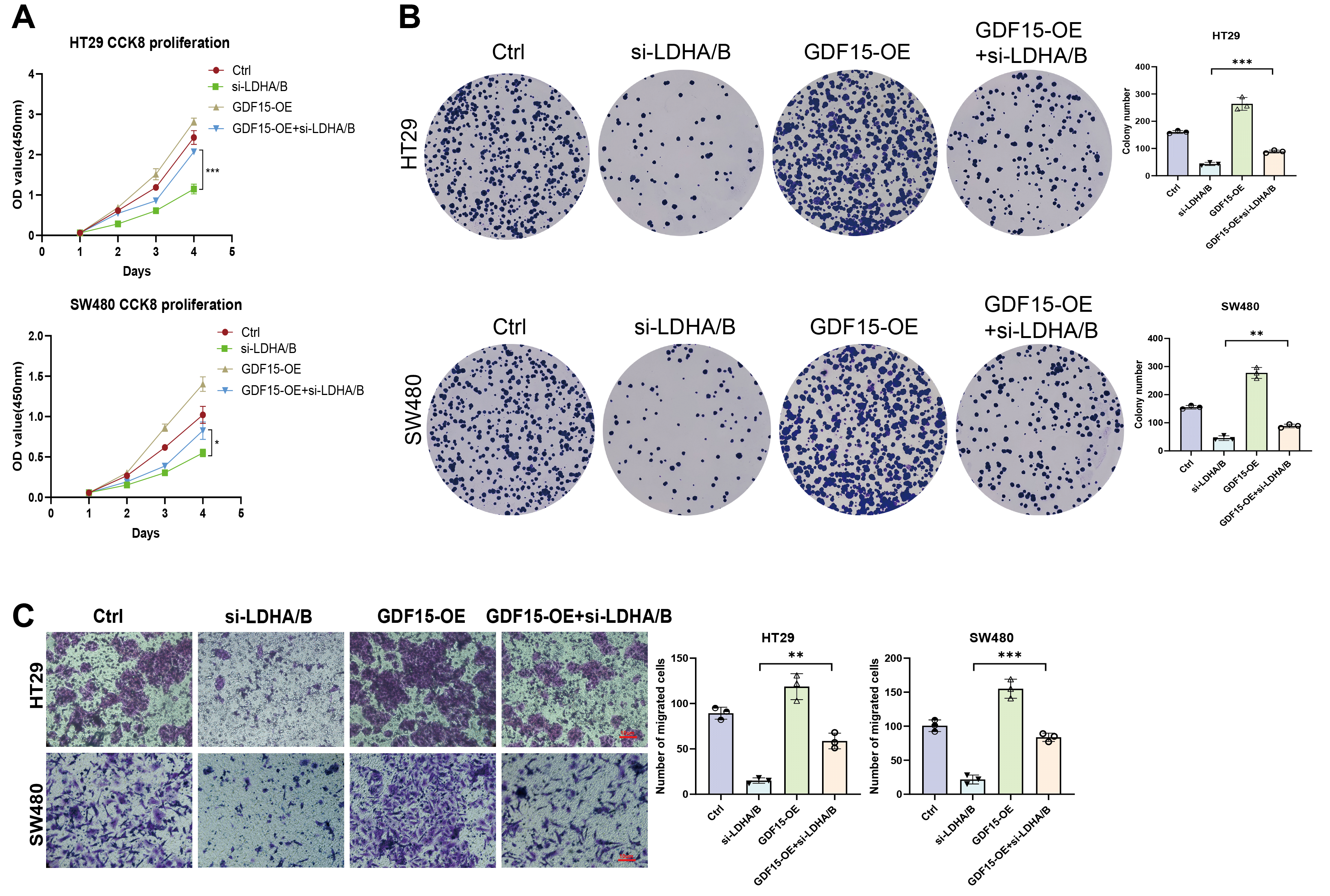

Supplement: Supplementary file 7 — Supplementary Figure S6 [file 41419_2025_7914_MOESM7_ESM.png]

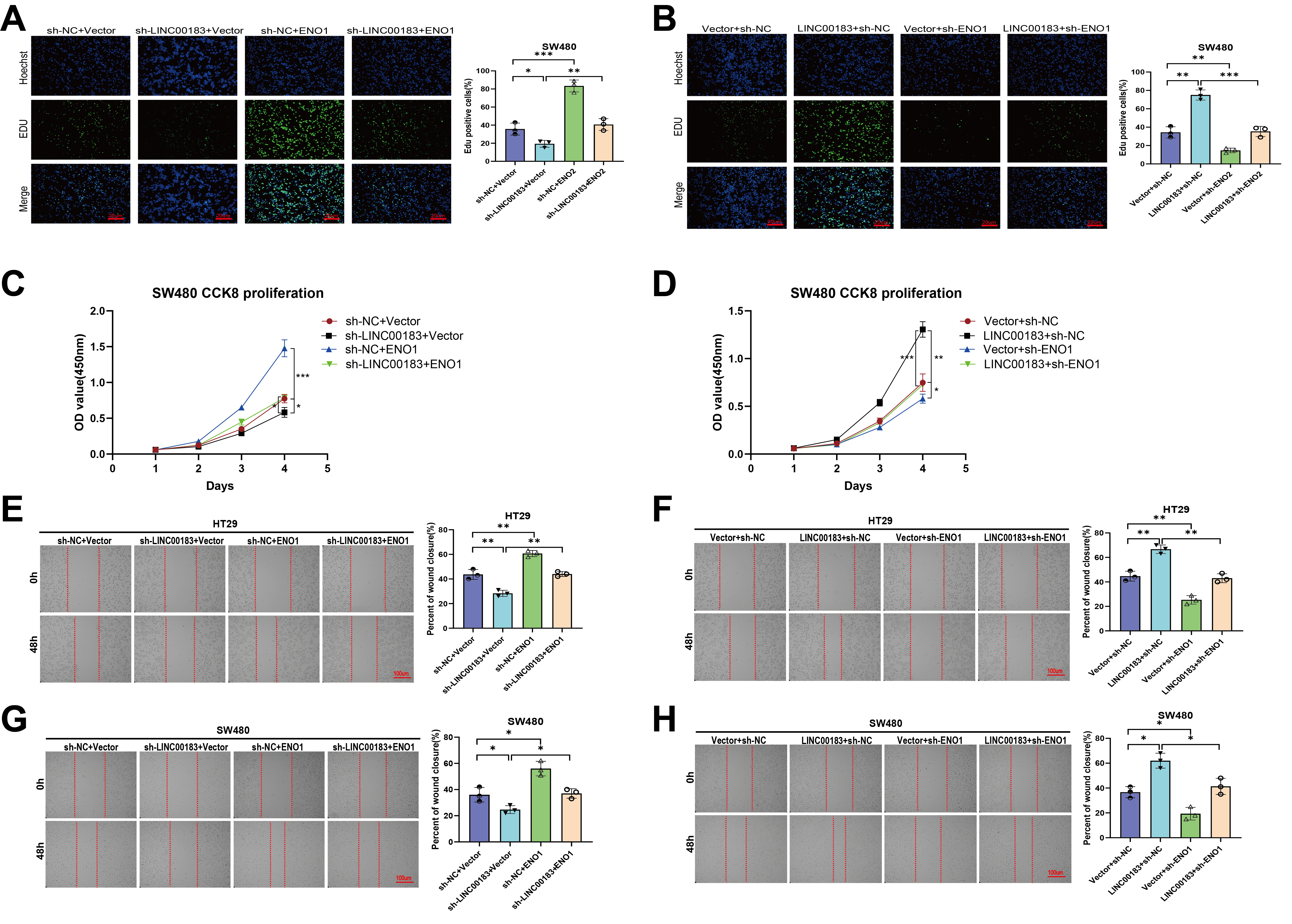

Supplement: Supplementary file 8 — Supplementary Figure S7 [file 41419_2025_7914_MOESM8_ESM.png]

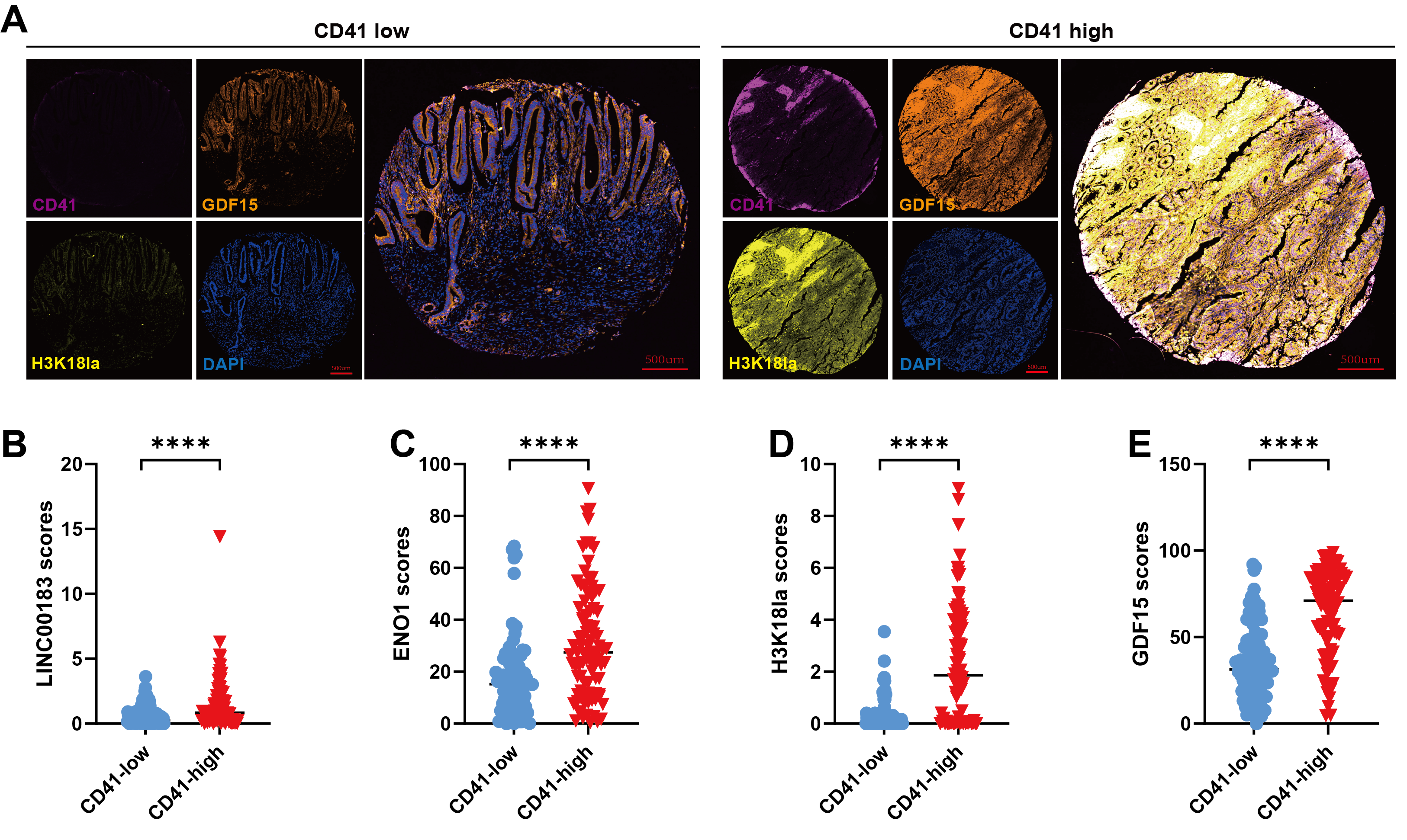

Supplement: Supplementary file 9 — Supplementary Figure S8 [file 41419_2025_7914_MOESM9_ESM.png]
