# Supplementary material for: Platelet-derived exosomal LINC00183 facilitate colorectal cancer malignant progression driven by histone lactylation through stabilizing ENO1
Source: Cell Death Dis. 2025 Aug 7;16(1):593. doi: 10.1038/s41419-025-07914-4 (PMC12331901; doi:10.1038/s41419-025-07914-4)

Figure 1 C

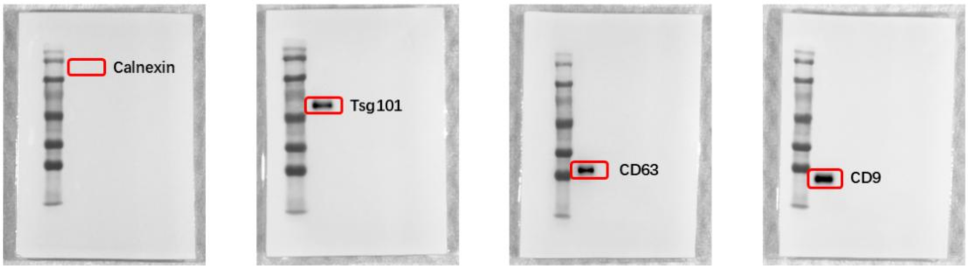

Figure 5 B

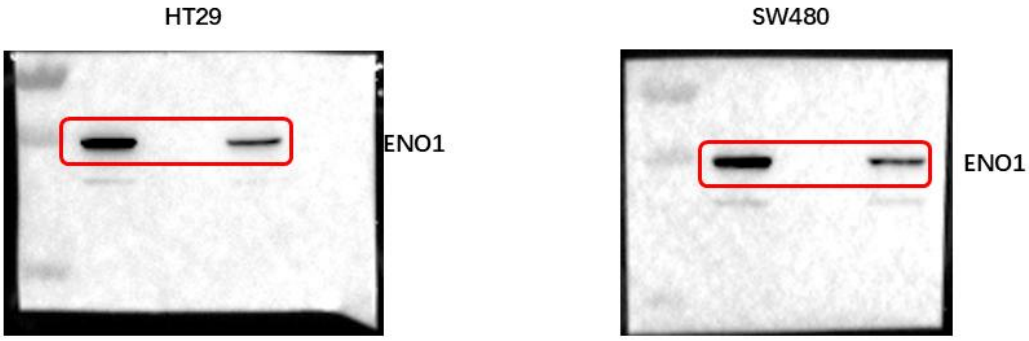

Figure 5 E

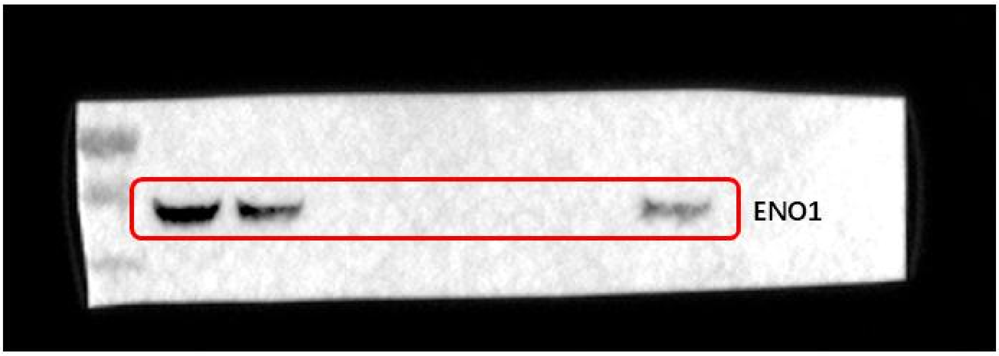

Figure 5 G

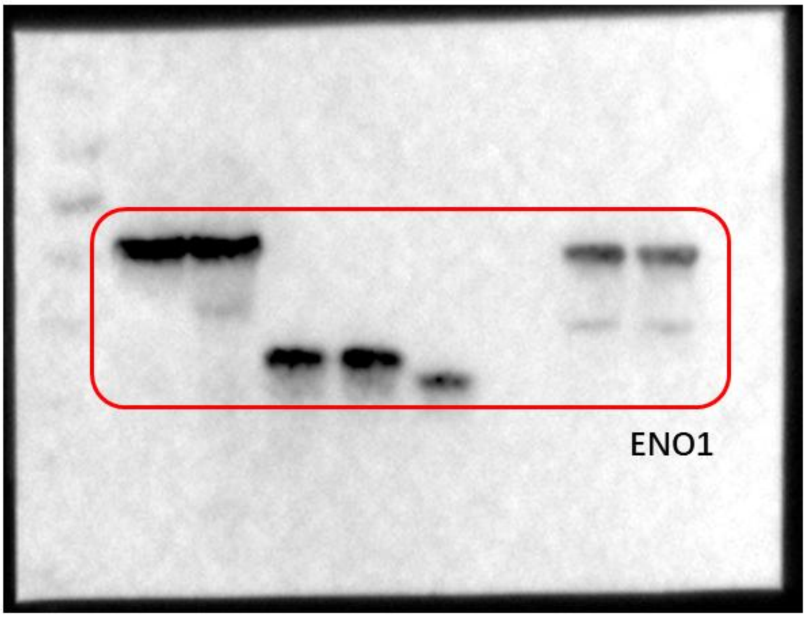

Figure 6 A

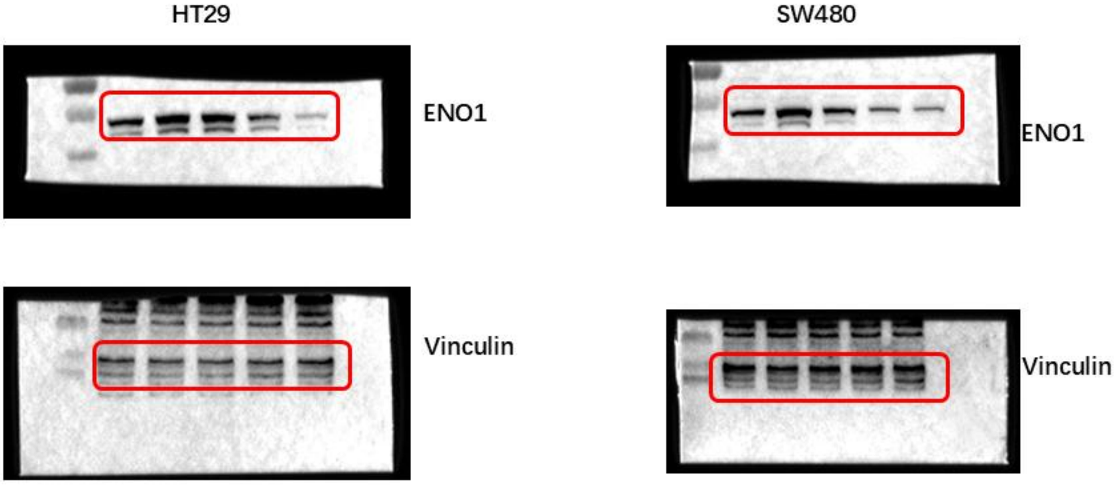

Figure 6 C

HT29

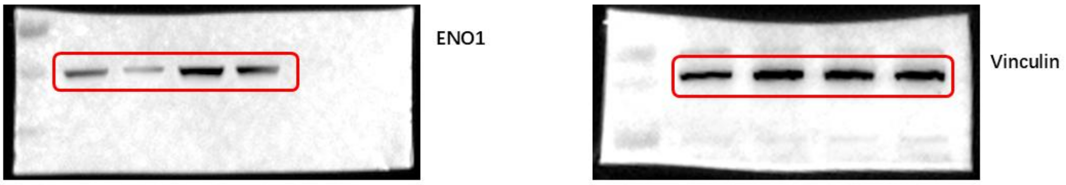

Figure 6 D

SW480

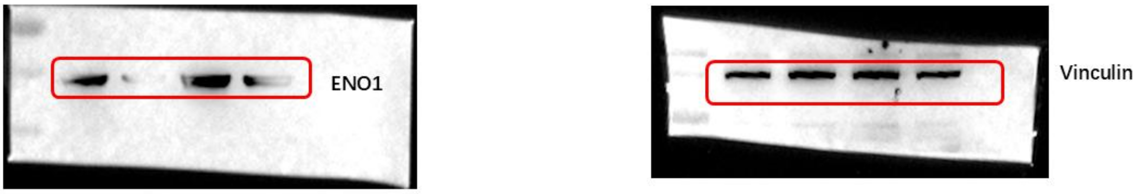

Figure 6 E

HT29

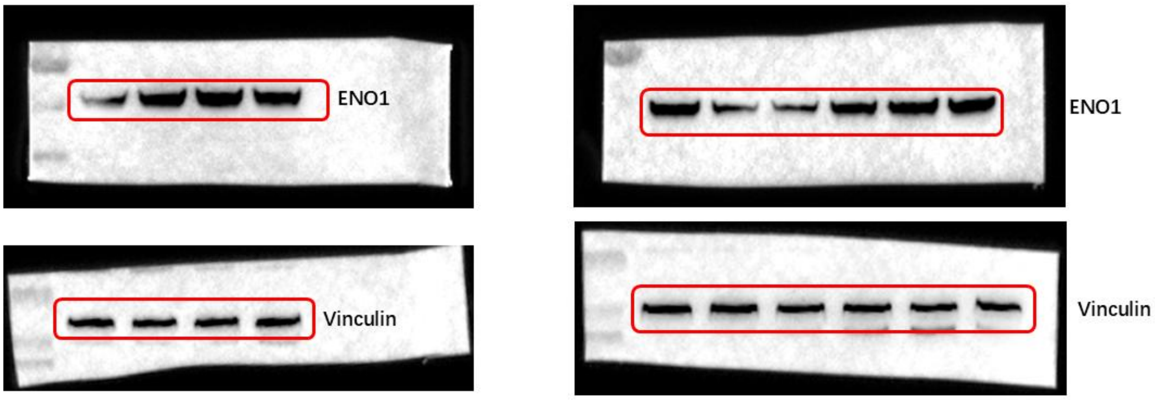

Figure 6 F

SW480

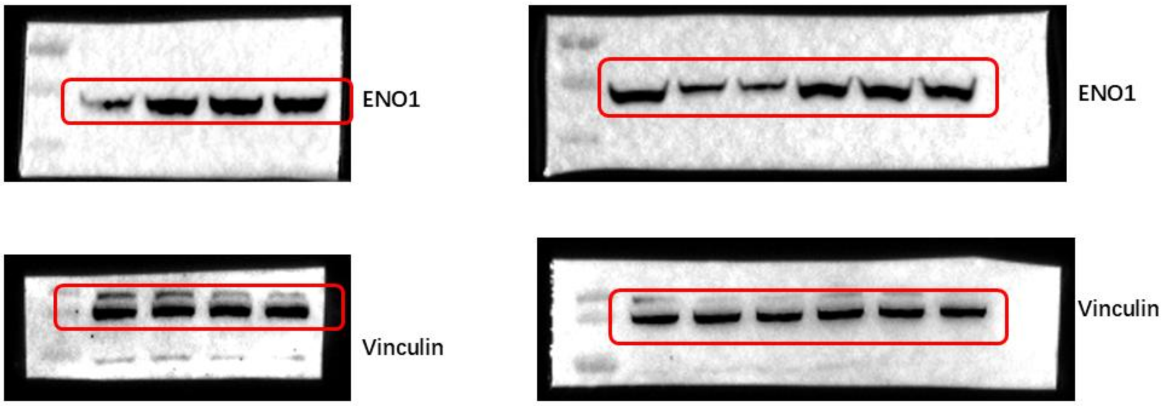

Figure 6 G

HT29

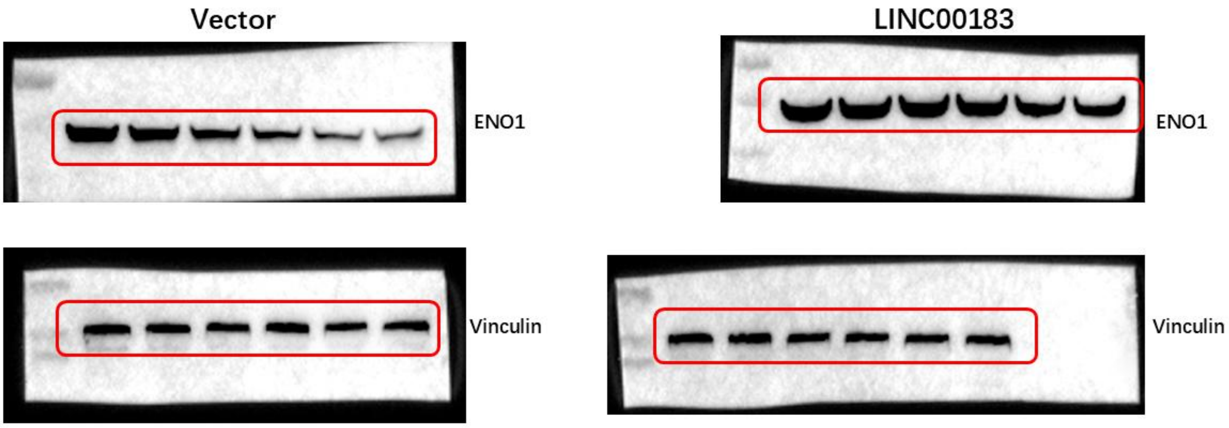

Figure 6 H

HT29

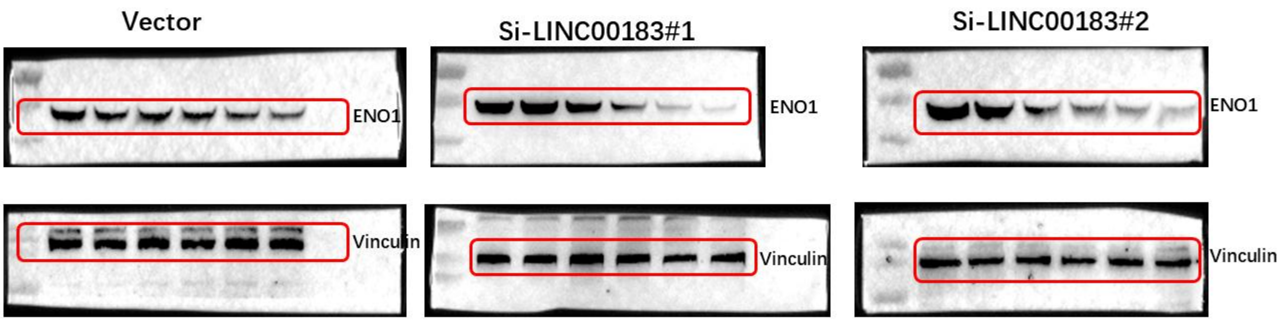

Figure 6 I

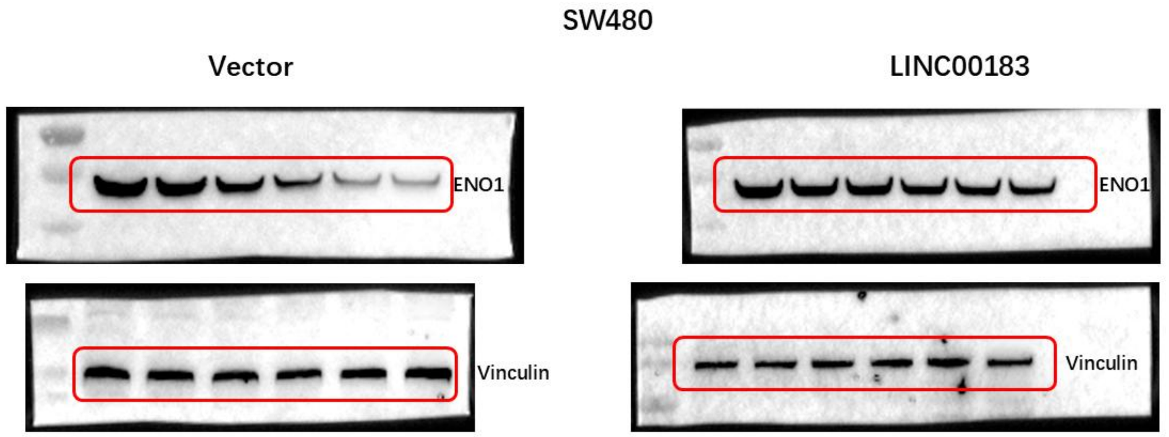

Figure 6 J

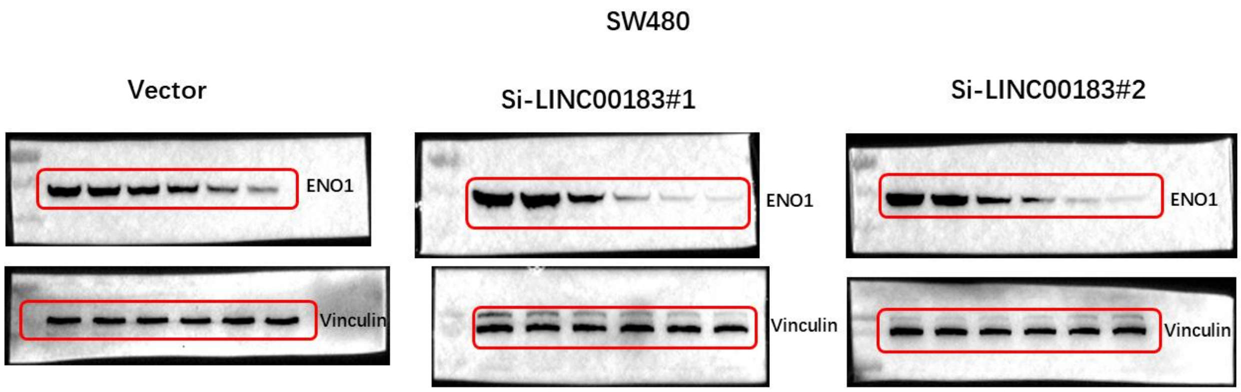

Figure 6 O

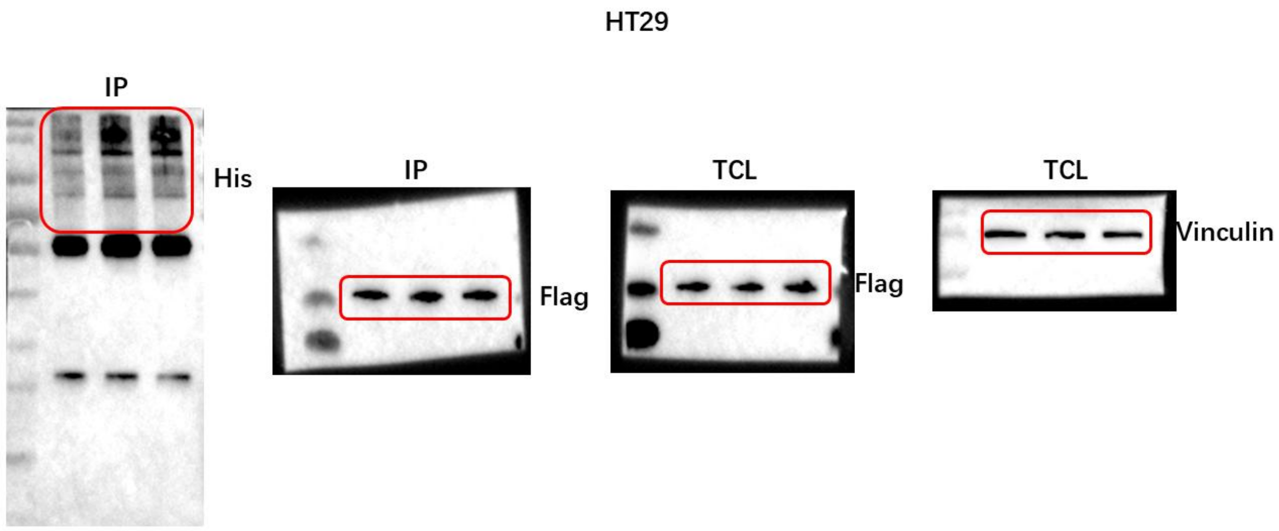

Figure 6 P

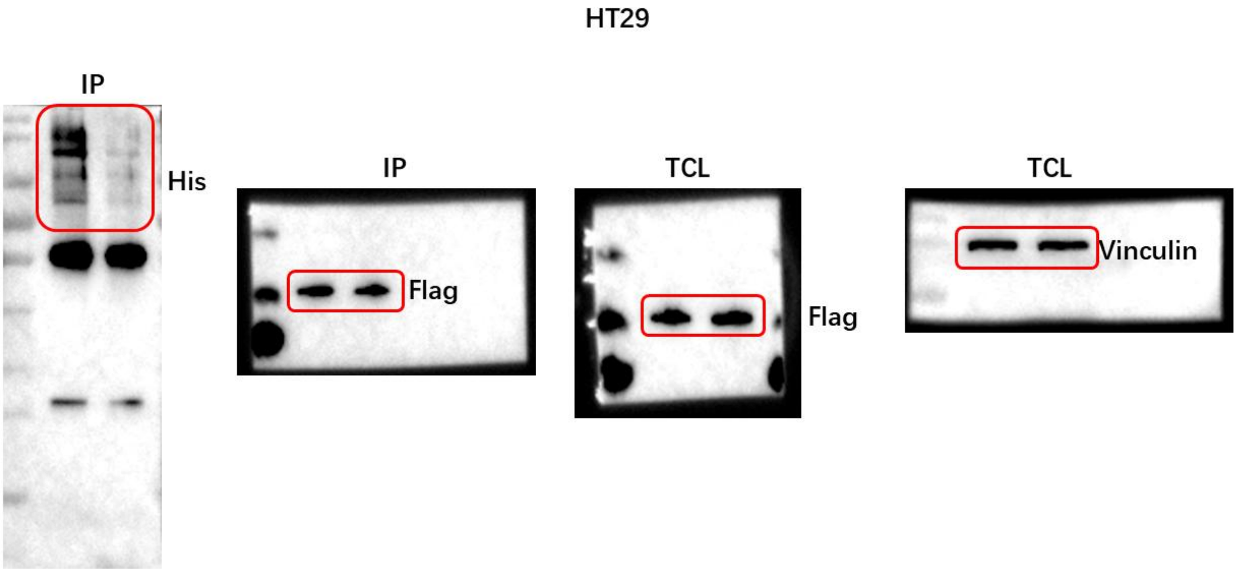

Figure 6 Q

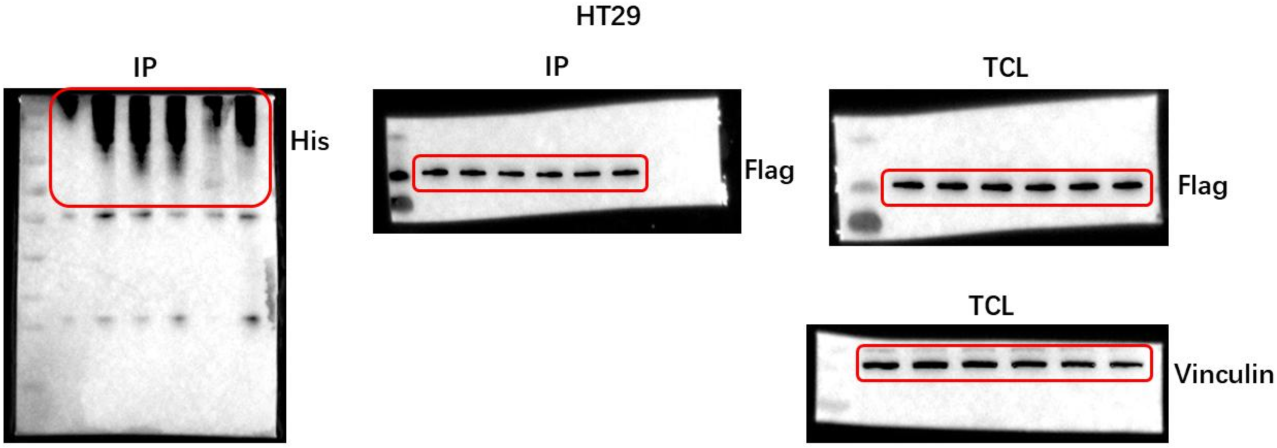

Figure 6 R

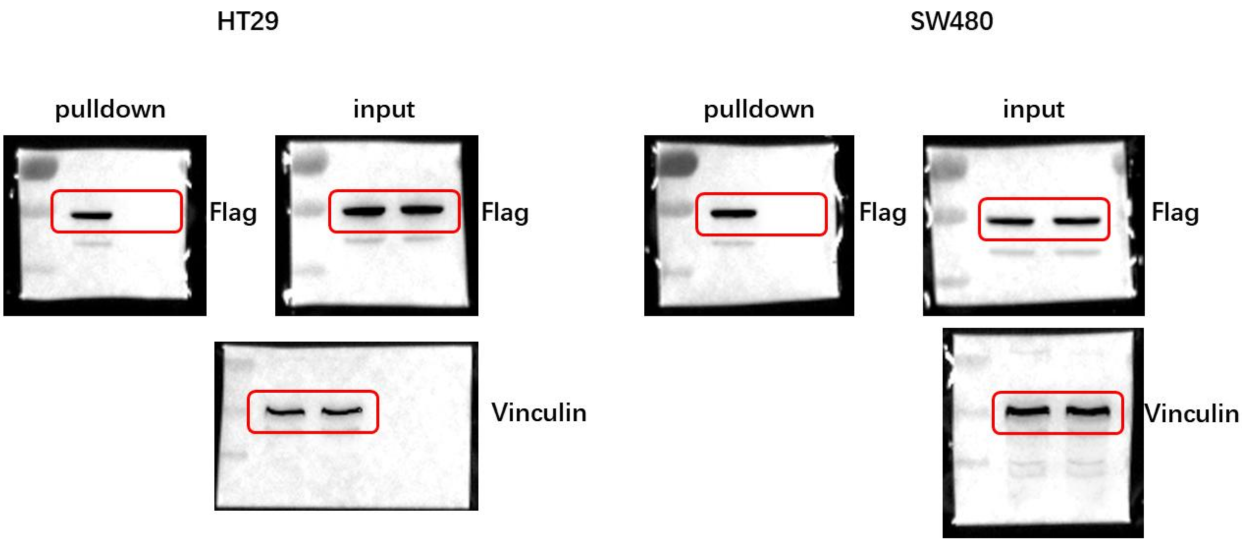

Figure 7 E

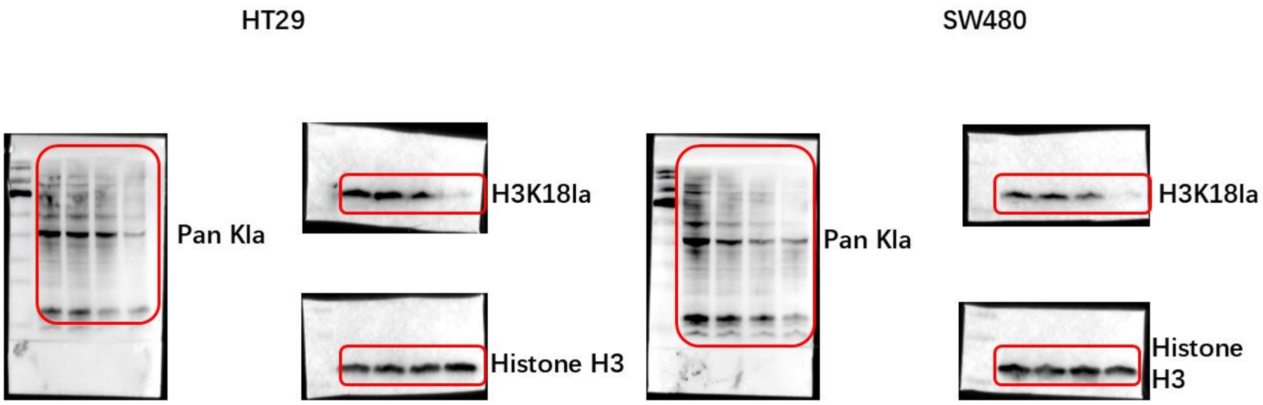

Figure 7 F

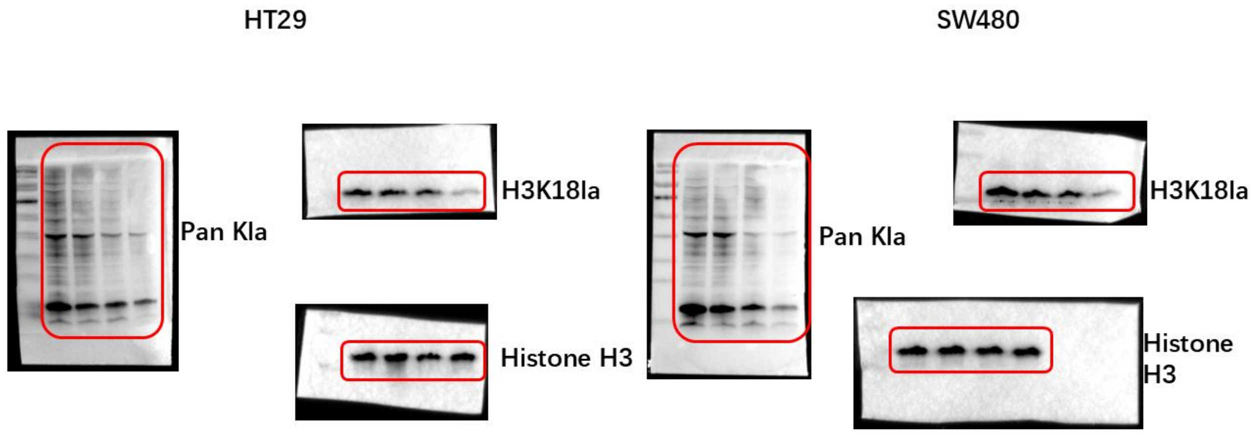

Figure 7 G

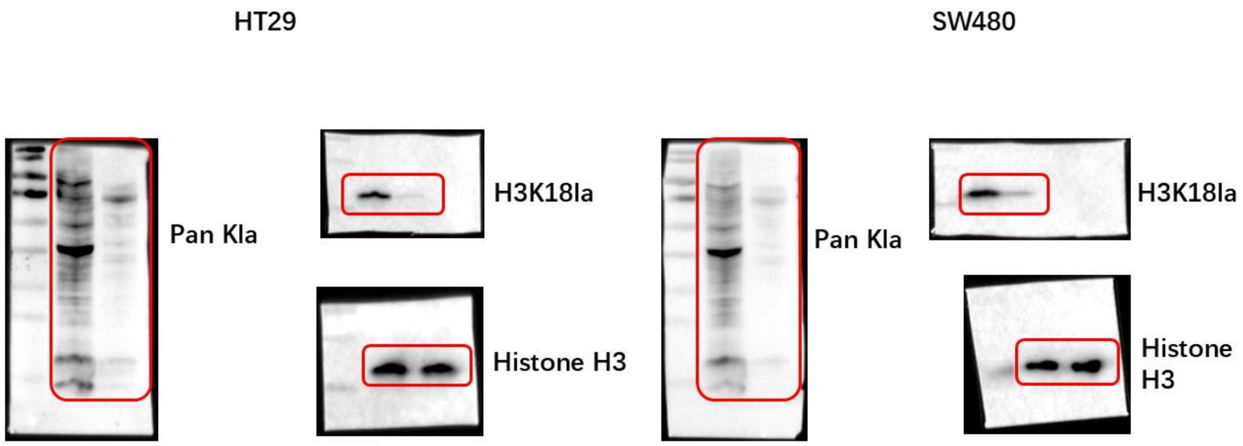

Figure 7 H

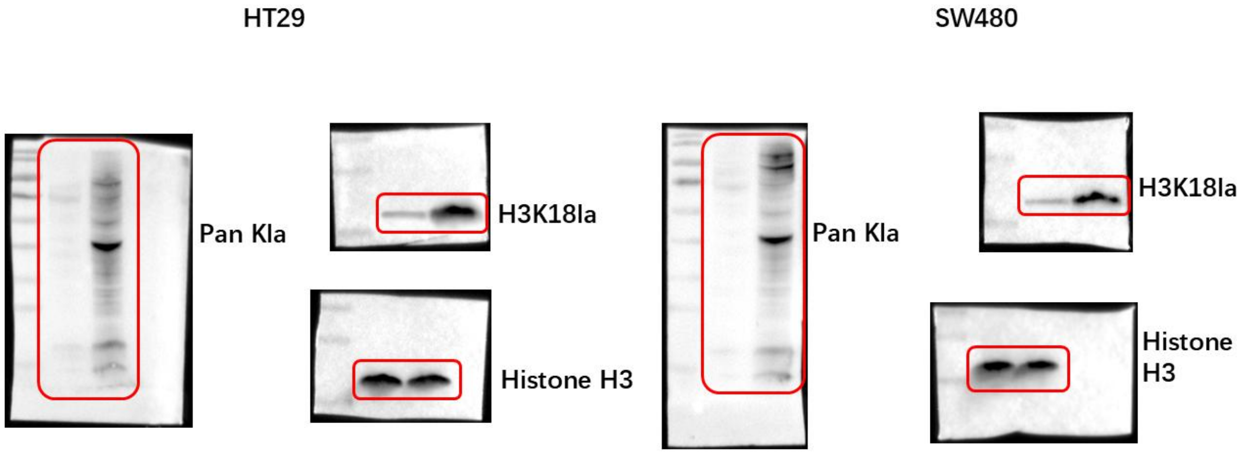

Figure 7 I

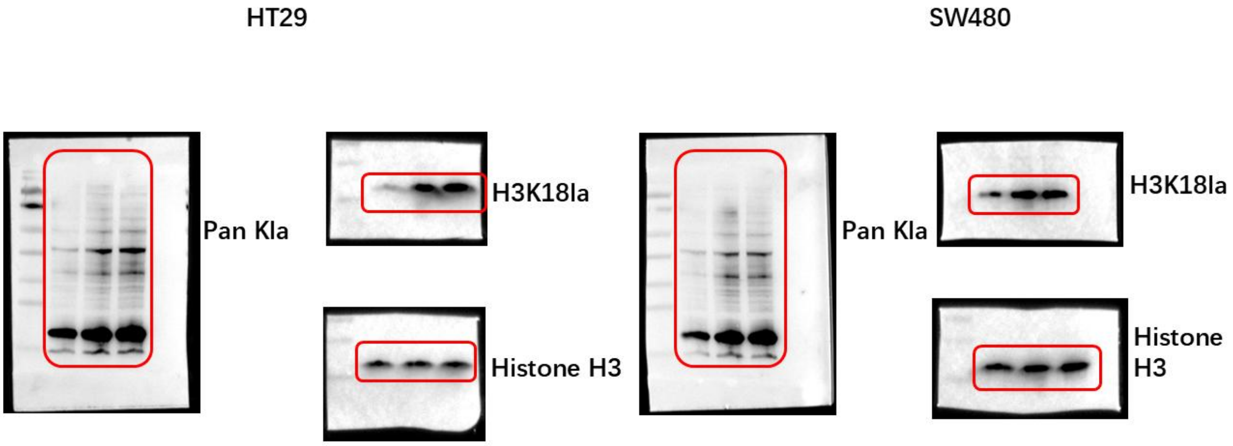

Figure 7 J

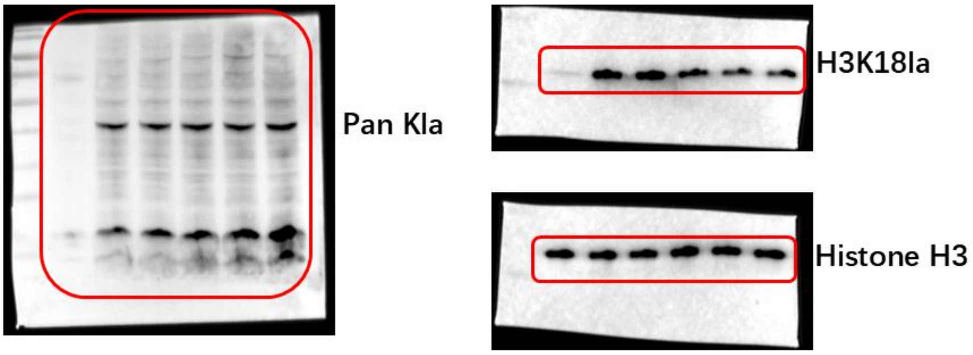

Figure 7 O

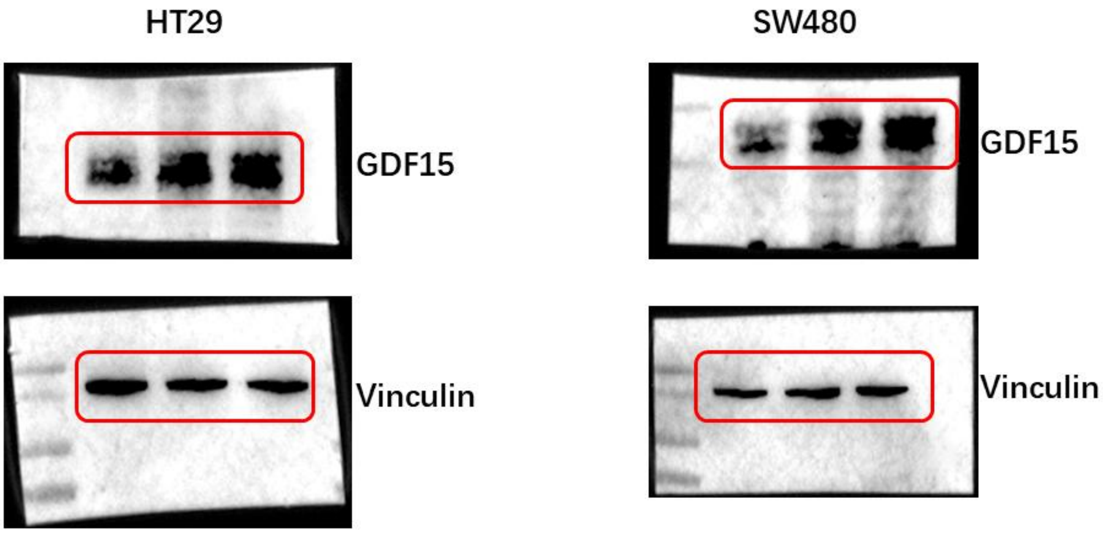

Figure 7 P

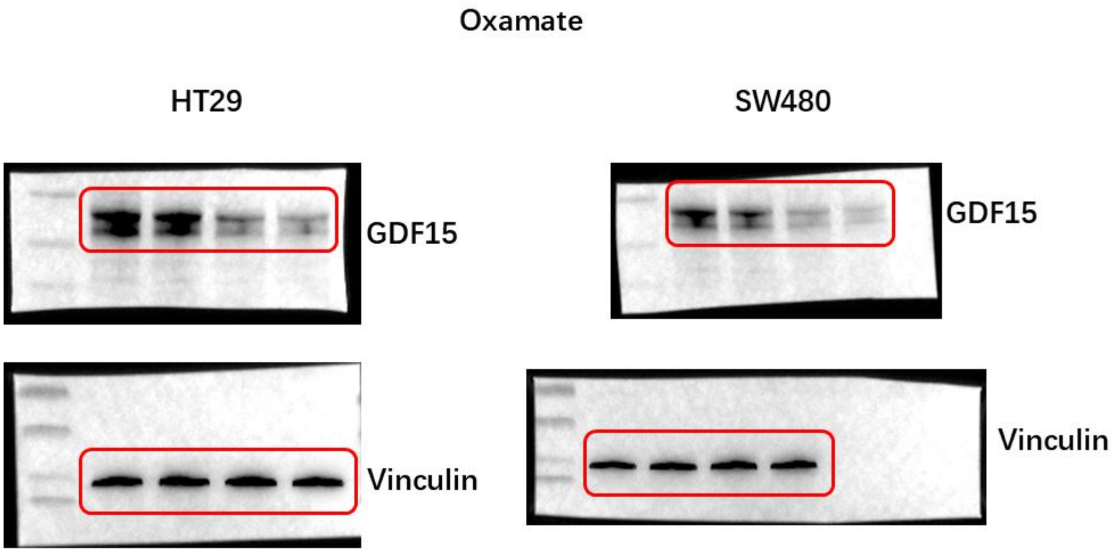

Figure 7 P

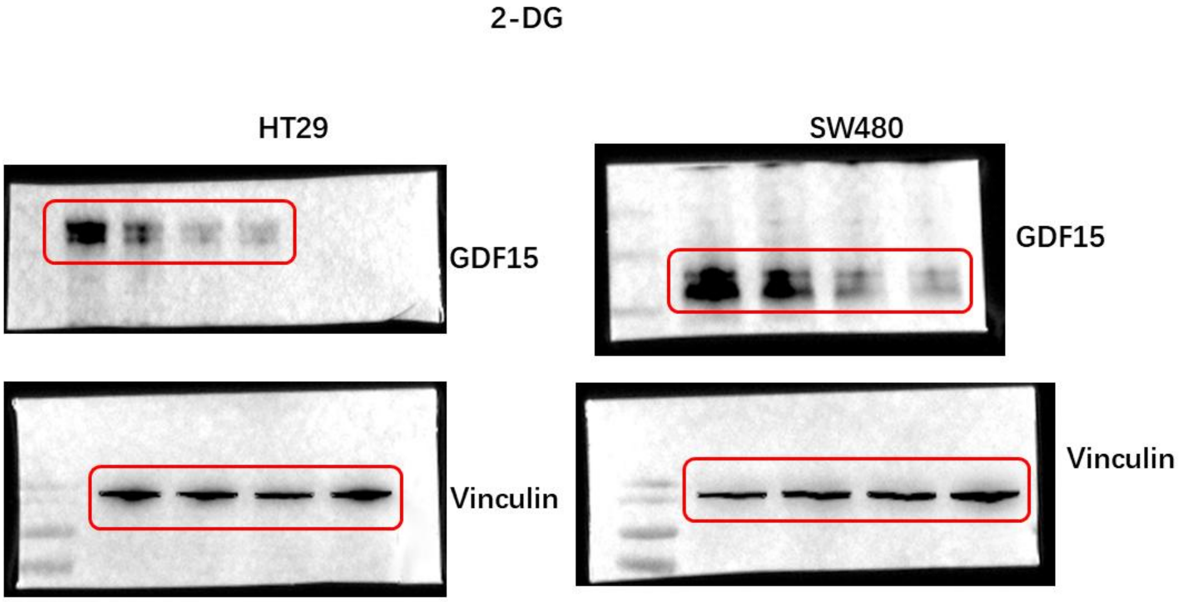

Figure 8 T

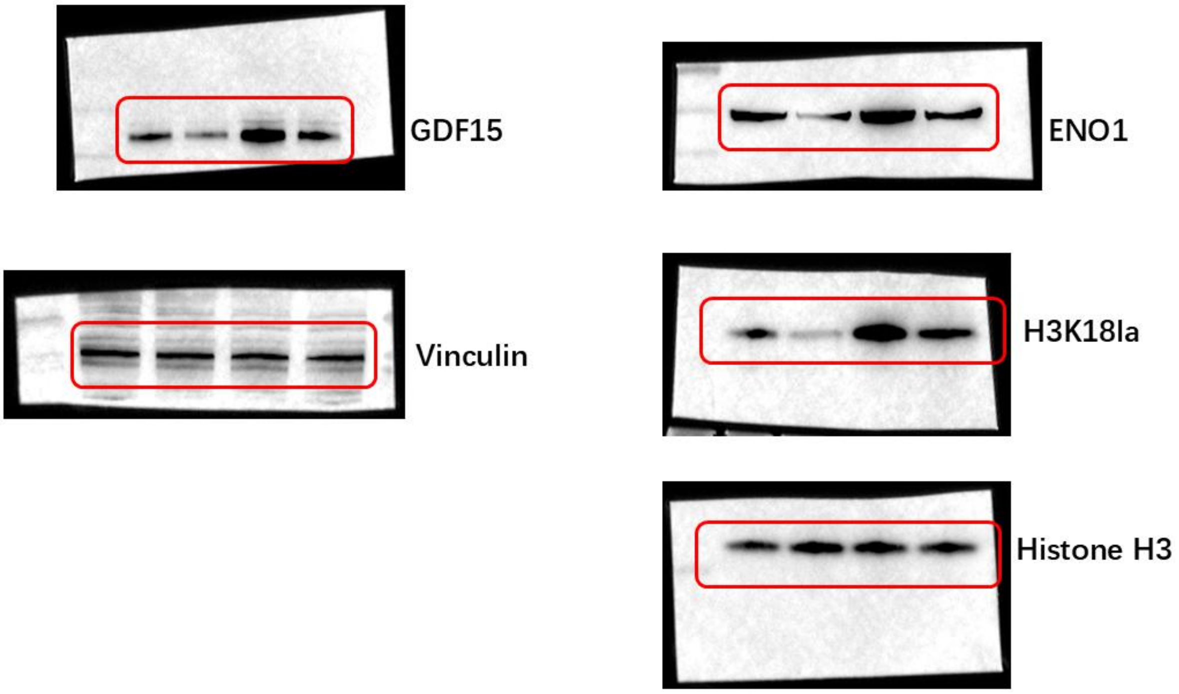

Figure 8 X

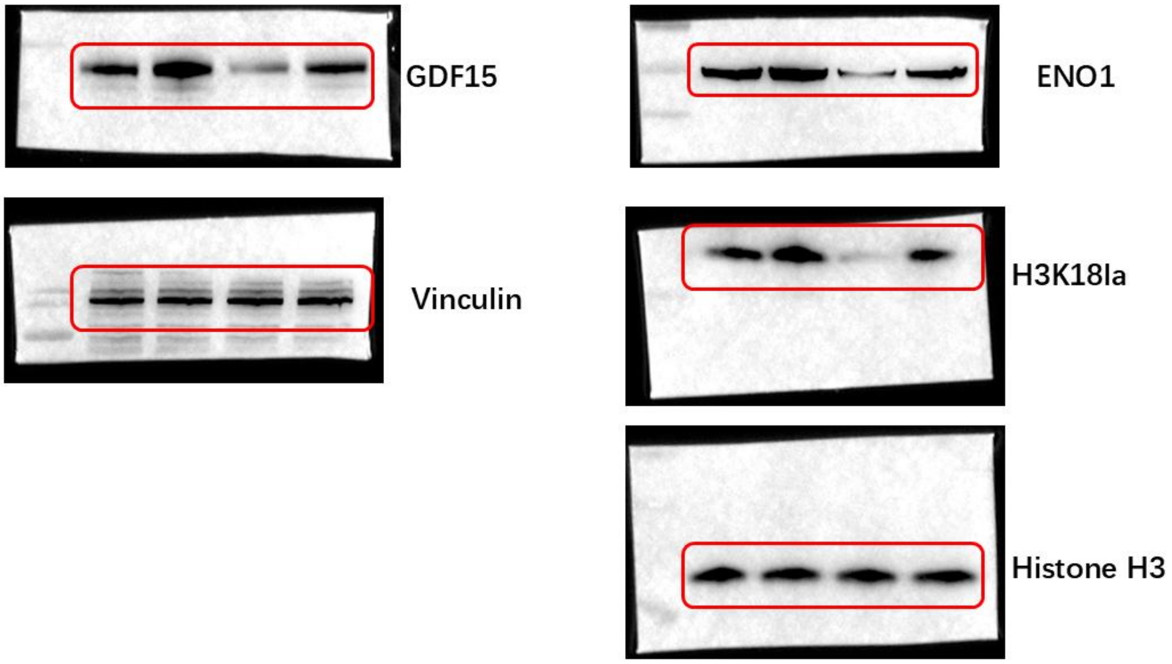

Supplementary Figure S2D

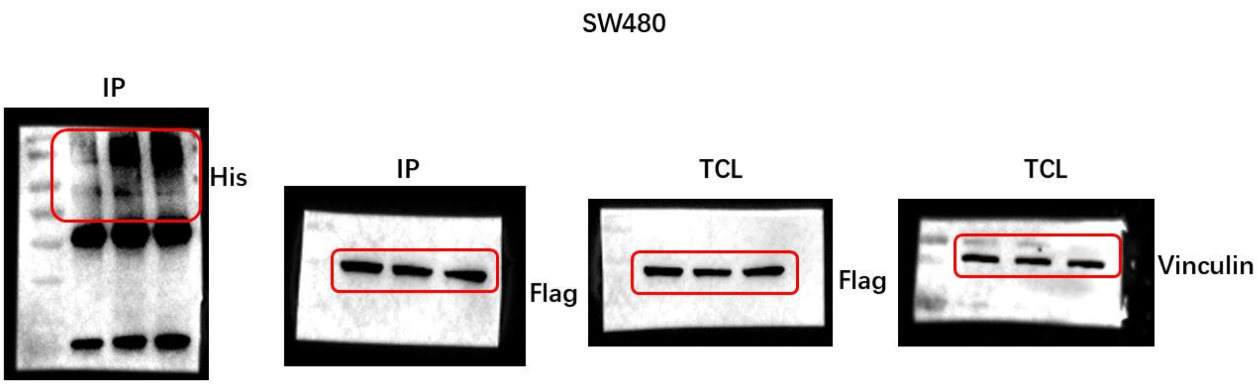

Supplementary Figure S2E

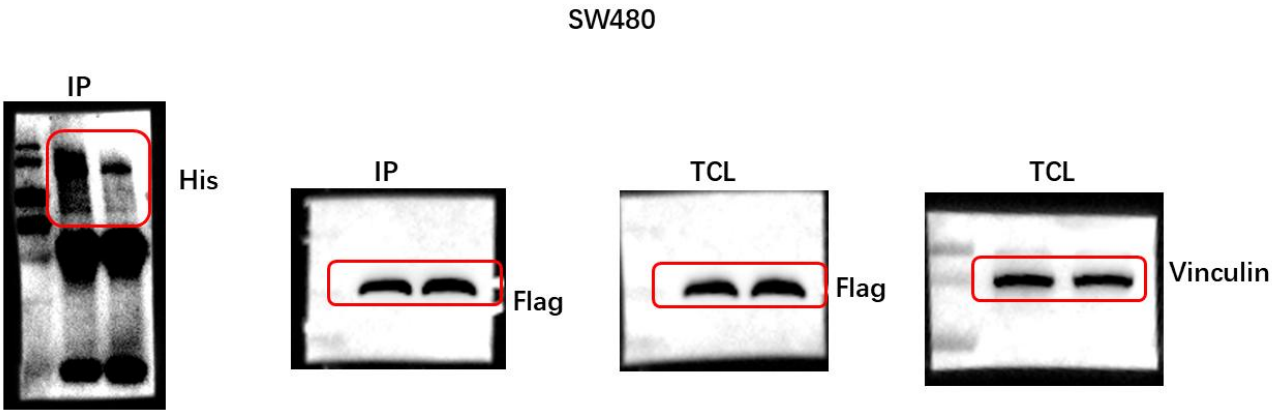

Supplementary Figure S2F

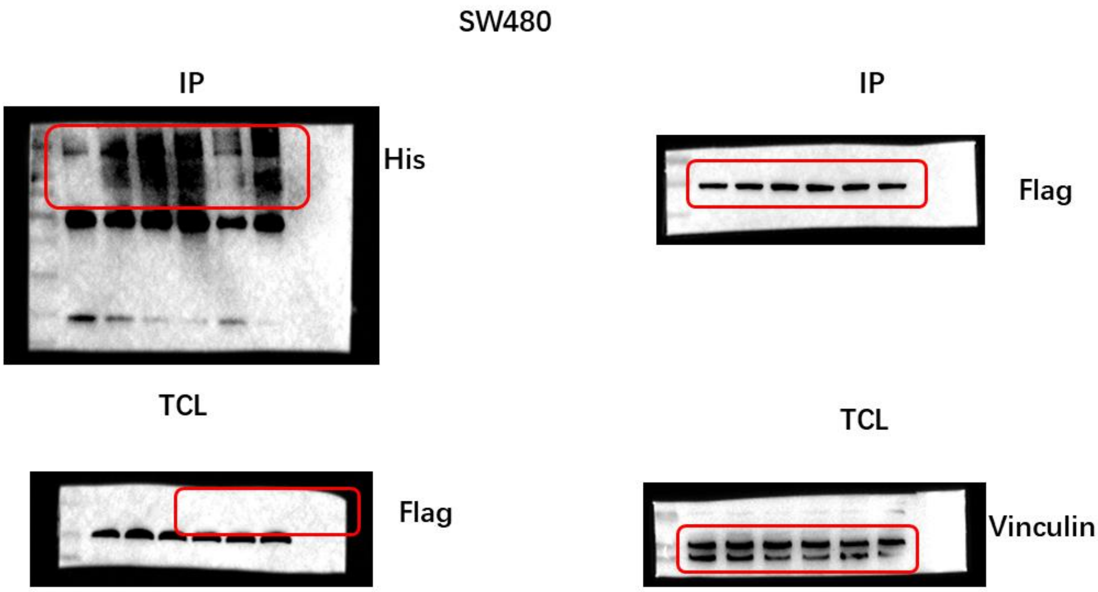

Supplementary Figure S2G

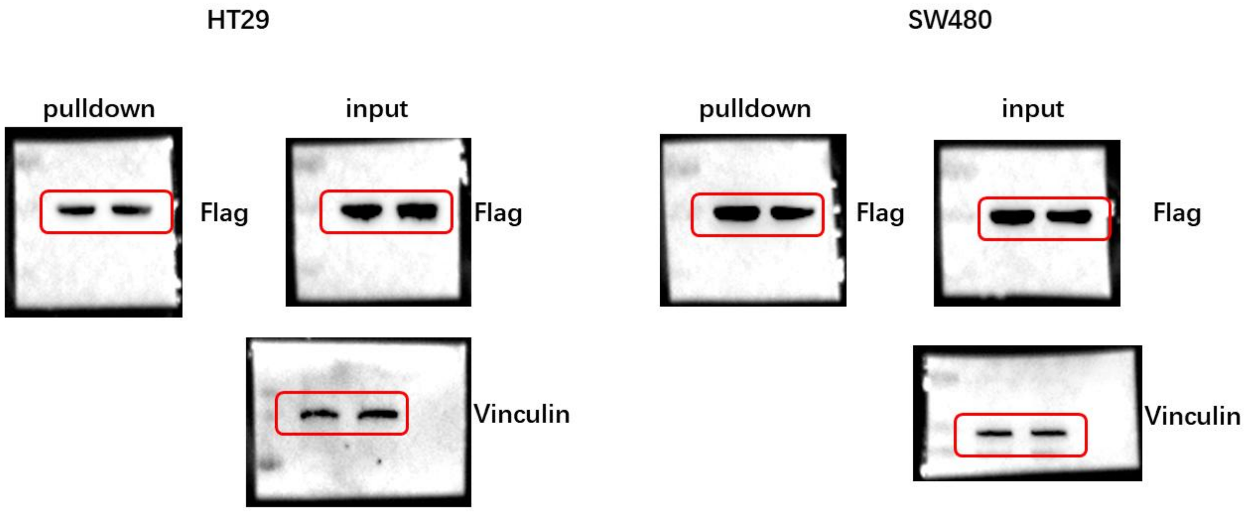

Supplementary Figure S2H

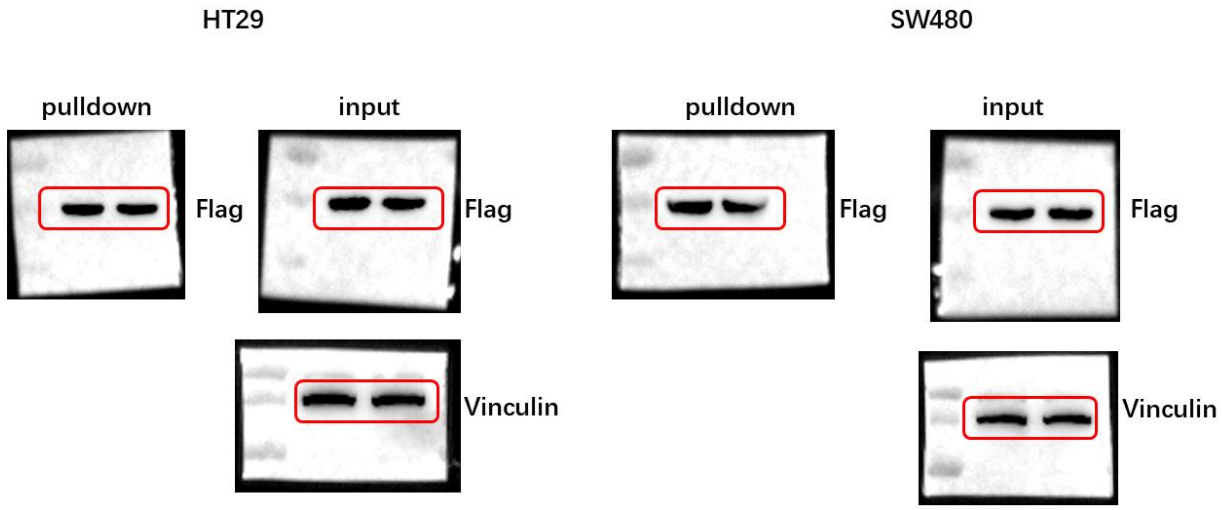

Supplementary Figure S2I

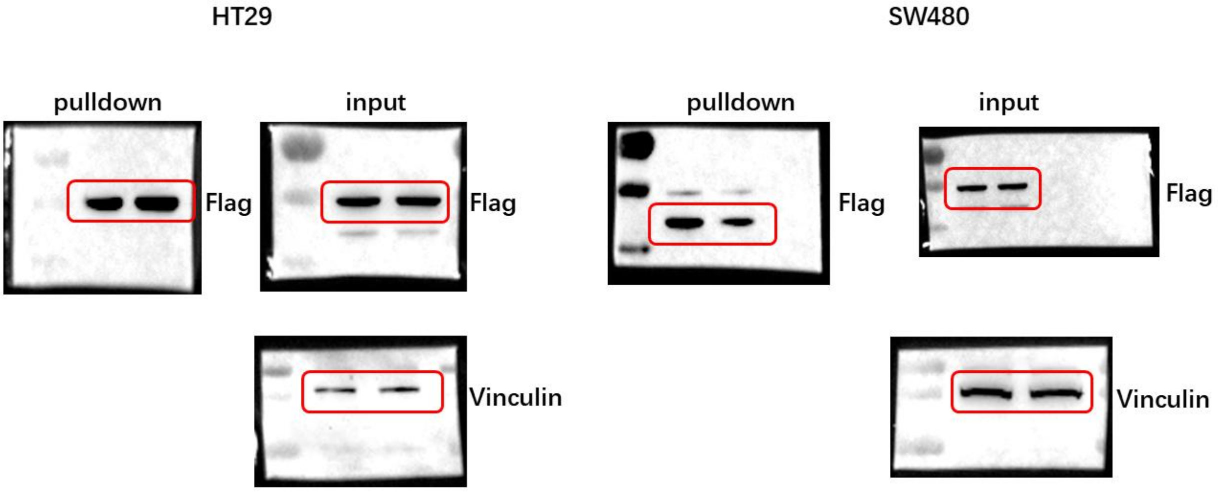

Supplementary Figure S4A

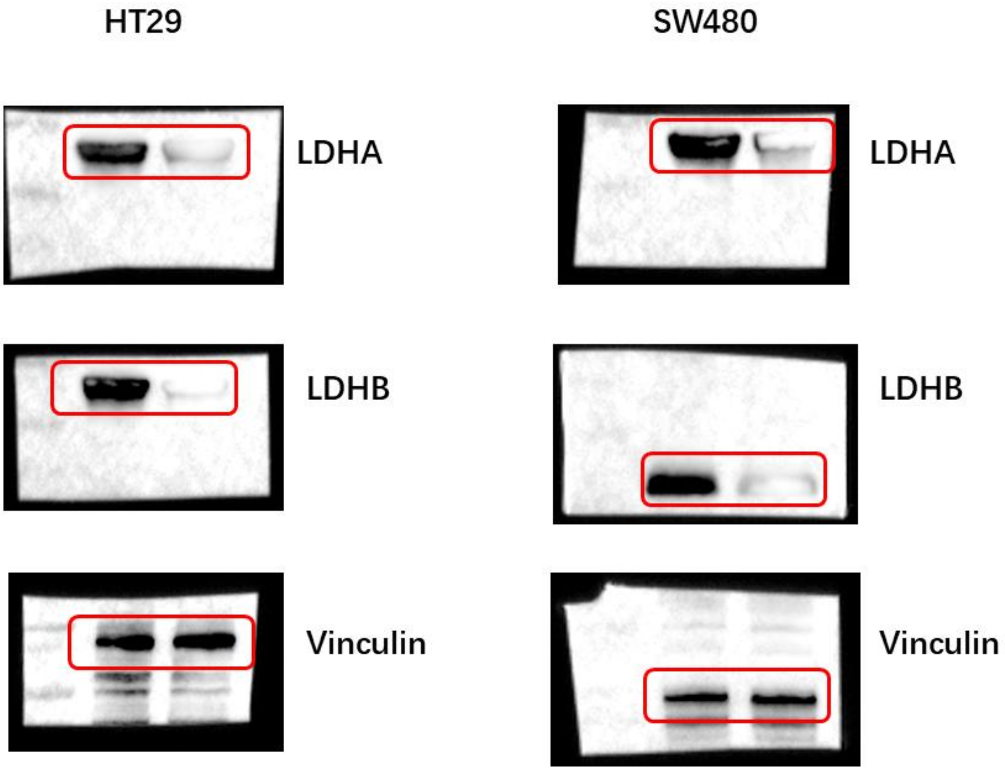

Supplement: Supplementary file 10 — WB-raw data [file 41419_2025_7914_MOESM10_ESM.pdf]
